# Differential *FGFR1* copy number status displays unique tumour immune microenvironment in triple‐negative breast cancer

**DOI:** 10.1002/path.70121

**Published:** 2026-09-09

**Authors:** Yike Gao, Fei Yao, Junyi Pang, Tianlin Sun, Changbin Zhu, Shafei Wu, Zhiyong Liang

**Affiliations:** ^1^ Department of Pathology, Molecular Pathology Research Centre Peking Union Medical College Hospital, Chinese Academy of Medical Sciences and Peking Union Medical College Beijing PR China; ^2^ Department of Medicine Amoy Diagnostics Co., Ltd. Xiamen PR China

**Keywords:** triple‐negative breast cancer (TNBC), fibroblast growth factor receptor 1 (*FGFR1*), HER2, amplification, tumour microenvironment, immune cell

## Abstract

Fibroblast growth factor receptor 1 (*FGFR1*) amplification, a significant cancer alteration with both prognostic and therapeutic relevance, lacks a standardized definition. The relationship between *FGFR1* copy number (CN) variations and tumour microenvironment (TME) characteristics also remains poorly understood. This study defined *FGFR1* amplification and investigated TME features across *FGFR1* CN subtypes in triple‐negative breast cancer (TNBC) to guide precise treatment. We analysed 182 TNBC tumour specimens. *FGFR1* CN status was assessed using fluorescence *in situ* hybridization (FISH). Next‐generation sequencing (NGS) was performed on 88 samples to explore biological differences. Immunohistochemical (IHC) results for CD3, CD4, and CD8 along with clinical data were analysed. Samples were categorized into four groups based on the *FGFR1/*CEN8 ratio and the mean *FGFR1* signals per cell. Groups 1 (ratio ≥ 2.0, signals ≥ 4.0) and 2 (ratio ≥ 2.0, signals < 4.0) exhibited robust tumour proliferation, increased *FGFR* signalling, and an immunosuppressive TME. Conversely, group 4 (ratio < 2.0, signals < 4.0) showed significantly enhanced immune cell infiltration (e.g. CD8^+^ T cells, NK cells), supported by elevated CD3^+^/CD8^+^ T‐cell densities. Group 3 (ratio < 2.0, signals ≥ 4.0) was heterogeneous: tumours with *FGFR1* CN ≥ 5 resembled groups 1/2, while those with CN < 5 mirrored group 4. Thus, *FGFR1* amplification (*FGFR1*‐Amp) was defined as *FGFR1/*CEN8 ratio ≥ 2.0 or CN ≥ 5.0 (14.29% prevalence, 26/182). Remaining cases were *FGFR1*‐neutral (*FGFR1*‐Neu). Immune profiling revealed that *FGFR1*‐Amp & HER2‐low TNBC had reduced immune cell infiltration but heightened oncogenic signalling (including epithelial–mesenchymal transition, angiogenesis) compared with other subgroups. In contrast, *FGFR1*‐Neu tumours, especially HER2‐zero, displayed increased immune cell infiltration (NK cells, effector cells), MHC I/II molecules, activation markers, and elevated immune checkpoint expression (e.g. *PD‐L1*, *CTLA4*, *LAG3*). This study defines *FGFR1* amplification in TNBC and characterizes its TME. *FGFR1*‐Amp in HER2‐low TNBC correlates with an immunosuppressive environment, potentially hindering HER2‐ADC efficacy and suggesting combined *FGFR* inhibition. Conversely, *FGFR1*‐Neu & HER2‐zero tumours with immune activation may respond better to immunotherapy. © 2026 The Author(s). *The Journal of Pathology* published by John Wiley & Sons Ltd on behalf of The Pathological Society of Great Britain and Ireland.

## Introduction

The fibroblast growth factor receptor 1 (*FGFR1*) gene encodes a receptor tyrosine kinase that initiates downstream signalling pathways, including MAPK/ERK and PI3K/AKT, which are integral to cellular proliferation, survival, and epithelial–mesenchymal transition (EMT) processes [1, 2]. The copy number (CN) alteration of *FGFR1*, which promotes tumour growth and reshapes the immune landscape, has emerged as a critical oncogenic event and a potential target in triple‐negative breast cancer (TNBC) [2, 3]. *FGFR1* CN gain was previously observed in approximately 7–10% of breast cancer cases, with a notable prevalence in certain TNBC subtypes [1, 4]. Clinically, elevated *FGFR1* CN is associated with poor overall survival in TNBC patients [5, 6]. Preclinical studies have demonstrated that *FGFR1* signalling exacerbates the immunosuppressive tumour microenvironment through multiple mechanisms, which may represent a critical factor contributing to poor prognosis. In particular, *FGFR1* activation in cancer‐associated fibroblasts enhances their proliferation and promotes the secretion of vascular cell adhesion molecule 1 (VCAM‐1), thereby establishing physical and chemical barriers that hinder T‐cell infiltration [3]. Furthermore, *FGFR1* signalling upregulates the expression of immunosuppressive cytokines, such as interleukin‐10 (IL‐10) and transforming growth factor‐beta (TGF‐β), along with angiogenic factors such as vascular endothelial growth factor (VEGF), thereby promoting an environment that facilitates immune evasion [7, 8]. Despite significant advancements in understanding *FGFR1* amplification in TNBC, the relationship between *FGFR1* amplification and the TNBC immune microenvironment remains poorly characterized.

In spite of ongoing research, a universally accepted standard for defining *FGFR1* amplification remains elusive. The most common definition of *FGFR1* amplification requires either an *FGFR1/*centromere 8 (CEN8) ratio exceeding 2.0 or six or more *FGFR1* copies per tumour nucleus [9, 10, 11]. However, other studies use different criteria, such as more than five *FGFR1* signals per nucleus in > 50% of cancer cells or the presence of large gene copy clusters [12]. These inconsistent thresholds for copy number or ratios across studies result in varying reports of its prevalence and clinical significance [2]. Consequently, establishing consensus guidelines for *FGFR1* amplification testing remains an imperative unmet need in precision oncology. Therefore, by integrating genomic and transcriptomic analyses, this study seeks to develop a standardized framework for defining *FGFR1* amplification and to investigate the characteristics of the tumour microenvironment (TME) associated with different *FGFR1* CN alterations. This methodology aims to improve diagnostic precision and guide therapeutic strategies for cancers involving *FGFR1* modifications.

## Materials and methods

### Ethics approval and patient consent

This study was approved by the Institutional Review Board of Peking Union Medical College Hospital (approval number: ZS‐2834). Informed consent forms were obtained from all patients.

### Patient cohort and sample classification

The study encompassed 182 TNBC samples with sufficient archived tissue available for *FGFR1* fluorescence *in situ* hybridization (FISH) testing, collected between 2016 and 2018. Tissue microarrays (TMAs) were constructed from formalin‐fixed, paraffin‐embedded (FFPE) specimens and subsequently employed for *FGFR1* FISH analysis. All haematoxylin and eosin (H&E)‐stained slides from each case were reviewed to map intratumoural heterogeneity, including architectural patterns and geographical locations. For each case, two tissue cores, each measuring 1 mm, were extracted from representative tumour regions (Figure 1A). Clinicopathological data were systematically collected. Following the 2023 American Society of Clinical Oncology/College of American Pathologists (ASCO/CAP) guideline update [13], HER2‐low was defined as IHC 1^+^ or 2^+^/ISH‐negative, while HER2‐zero was defined as IHC 0.

**Figure 1:**
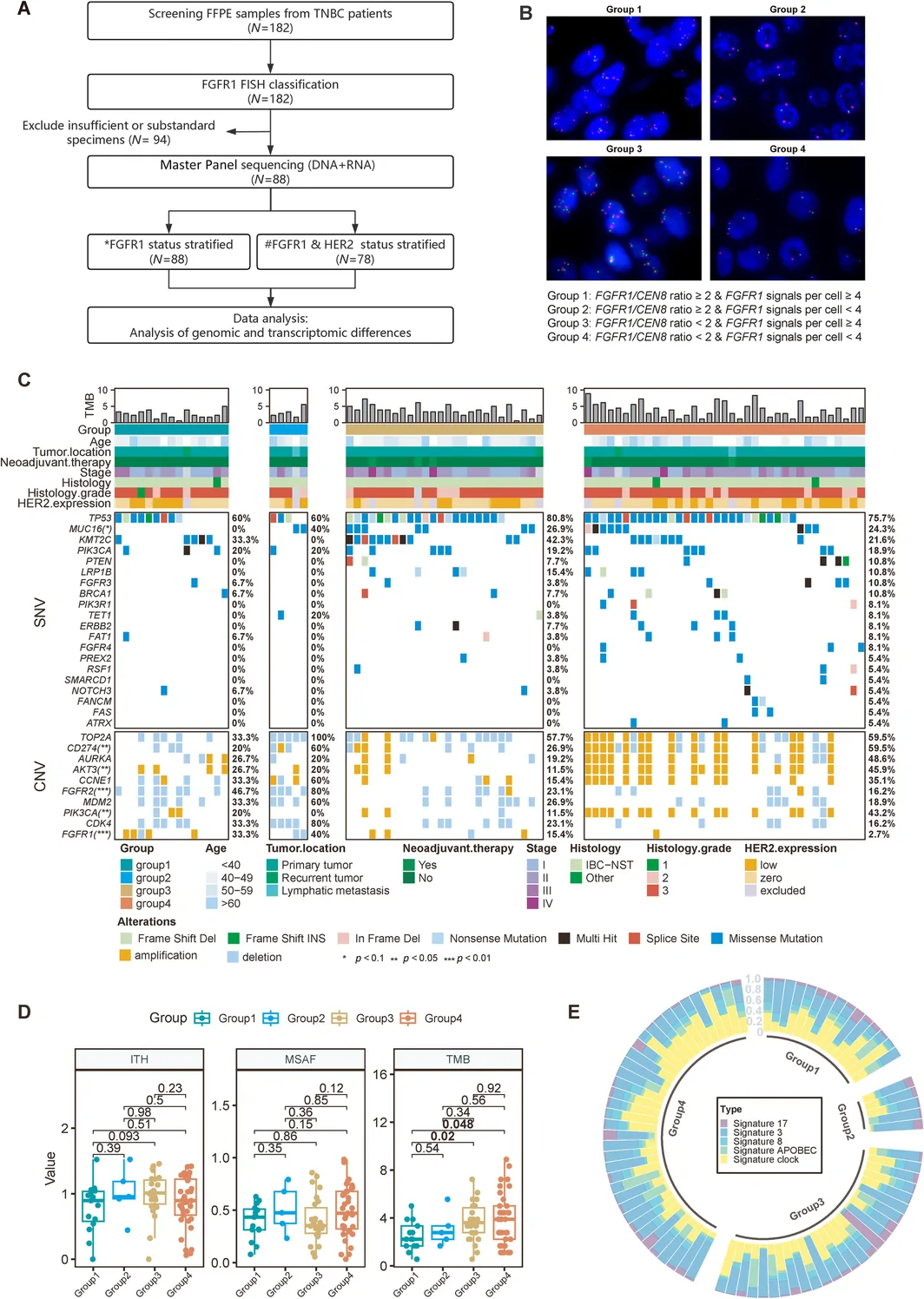
Comprehensive genomic and clinical landscape of *FGFR1*‐altered TNBC. (A) The workflow of this study. (B) Representative FISH images of *FGFR1* (red) and CEN8 (green). Samples were categorized into four distinct subtypes based on *FGFR1* fluorescence *in situ* hybridization analysis. Images were taken at 1,000× total magnification. (C) Genetic alterations and clinical characteristics in patient cohorts. (D) Correlation between intratumoural heterogeneity (ITH), mutant subclonal allele frequency (MSAF), tumour mutation burden (TMB), and *FGFR1* status. (E) Association between *FGFR1* status and mutation signatures. *p* values of statistical significance are shown in bold. TNBC, triple‐negative breast cancer; FISH, fluorescence *in situ* hybridization; CEN8, centromere of chromosome 8. *Stratified by *FGFR1* status alone (uses all 88 samples; *n* = 88). ^#^Stratified by both *FGFR1* and HER2 status (only primary tumours without neoadjuvant therapy were included, resulting in *n* = 78).

### *FGFR1* fluorescence *in situ* hybridization

The *FGFR1* FISH assays were conducted on 4‐μm‐thick FFPE tissue sections using an *FGFR1/CEN 8* Dual Color FISH Probe (Zyto*Light*; ZytoVision GmbH, Bremerhaven, Germany) following the manufacturer's protocol. The slides were scanned and analysed using an Olympus BX51 Fluorescence Microscope (Olympus, Shinjuku, Tokyo, Japan). Pictures were taken at 1,000× total magnification. For each tissue core, *FGFR1* (red) and CEN8 (green) signals were enumerated in at least 20 tumour cell nuclei by two independent observers. Given the lack of a universally accepted standard for defining *FGFR1* amplification, we adopted clinically validated HER2‐FISH criteria (ASCO/CAP guidelines) [13] as a reference framework for cohort stratification, combined with evidence from previous reports [12, 14]. We identified an *FGFR1/*CEN8 ratio of 2.0 and *FGFR1* signals per cell of 4.0 and 5.0 as key cutoff points. For cases with values approaching these cutoffs, a re‐evaluation was performed, with at least 20 additional tumour cell nuclei recounted to ensure accuracy.

### Immunohistochemical staining

Immunohistochemical staining was performed on 4‐μm paraffin sections using a Leica BOND‐III automated immunostainer (Leica, Bannockburn, IL, USA) or Dako Autostainer Link 48 (Agilent Technologies, Santa Clara, CA, USA). Primary antibodies were applied in accordance with the manufacturers' protocol, including CD3, CD4, CD8, AR, FOXC1, and DCLK1 (supplementary material, Table S1). Densities of CD3^+^, CD4^+^, and CD8^+^ tumour‐infiltrating lymphocytes per mm^2^ were calculated using Fiji (version 1.53c), an open‐source distribution of ImageJ, available at https://imagej.net/software/fiji/ (accessed 20 August 2024). TNBC subtyping was determined by immunohistochemistry for AR, CD8, FOXC1, and DCLK1. A cutoff of ≥ 10% positive tumour cells was used to define positivity for AR, FOXC1, and DCLK1, while CD8 positivity was defined as ≥ 20% positive cells among all nucleated cells. Based on these markers, TNBCs were classified as luminal androgen receptor (LAR: AR^+^), immunomodulatory (IM: AR^−^/CD8^+^), basal‐like immune suppressed (BLIS: AR^−^/CD8^−^/FOXC1^+^), mesenchymal‐like (MES: AR^−^/CD8^−^/FOXC1^−^/DCLK1^+^), or unclassifiable (UC: negative for all four markers) [15, 16].

### Next‐generation sequencing

Nucleic acid was extracted from six to eight tissue sections (4–5 mm thick) of FFPE samples, depending on the tumour content for each of the cases. DNA was extracted using a QIAamp DNA FFPE Tissue Kit (QIAGEN, Hilden, Germany), while RNA was extracted using an RNeasy FFPE Kit (QIAGEN), following dewaxing of the tissue sections. Nucleic acid was quantified using a NanoDrop One Spectrophotometer (BioTek Instruments, Winooski, VT, USA), and the quality was assessed using a Quantus Fluorometer (Promega, Madison, WI, USA). DNA was fragmented into 200–250 bp fragments and then underwent procedures including indexing, end‐repairing, A‐tailing, adaptor ligation, and amplification using the NEBNext Ultra II DNA Library Prep Kit (New England BioLabs, Ipswich, MA, USA). RNA was fragmented, followed by reverse transcription, cDNA synthesis, and strand‐specific library preparation using the NEBNext Ultra II Directional RNA Library Prep Kit for Illumina. DNA and RNA libraries were captured separately using the AmoyDx Master Panel containing 571 genes for DNA‐based analyses (single nucleotide variant, indel, fusion, copy number variation, microsatellite instability, and tumour mutation burden) and 2,660 genes for RNA expression and fusion detection (supplementary material, Tables S2–S5). Library sequencing was performed on an Illumina NovaSeq 6000 sequencer (Illumina, Inc., San Diego, CA, USA), generating 2 × 150 bp paired‐end reads.

### Genomic variation analysis

Intratumour heterogeneity (ITH), maximum somatic allele frequency (MSAF), and tumour mutation burden (TMB) were compared across different groups. To delineate the mutational signatures of gene mutations, we used the SigMA (v 1.6) R package, which is a likelihood‐based measure combined with machine‐learning techniques [17].

### Differential gene expression and pathway analysis

The Wilcoxon rank‐sum test, a classical non‐parametric statistical test, was used to compare gene expression levels between two conditions. Differentially expressed genes (DEGs) were identified based on the following criteria: genes with fold‐change ≥ 1.5 and *p* value < 0.05 for the comparison between *FGFR1* groups. Enrichment analyses were conducted using the R package ‘clusterProfiler’ to determine the functional roles of DEGs. These included Gene Ontology (GO), Kyoto Encyclopedia of Genes and Genomes (KEGG), Hallmark, WikiPathways, and Reactome. Gene set enrichment analysis (GSEA) was also performed by the R package ‘GSEA’.

### Tumour microenvironment analysis

The gene set variation analysis (GSVA) algorithm was used to evaluate the relative abundance of infiltrating immune cells in the TME. Gene sets for TME‐infiltrating immune cells were extracted from previous datasets [18]. Enrichment scores calculated using the R package ‘GSVA’ were used to determine the relative abundance of each TME‐infiltrating cell [19]. The deconvolution method CIBERSORT [20] and marker‐based approach MCP‐counter [21] were also used to assess the TME.

### Statistical analyses

Statistical analyses were performed using R software version 4.1.2 (https://www.r-project.org/, accessed 10 February 2022), GraphPad Prism version 9.0.0 (GraphPad Software, LLC, Boston, MA, USA), and SPSS version 27.0.1 (IBM Corp., Armonk, NY, USA). Fisher's exact test was utilized to compare unordered categorical variables. For continuous variables, we used the Mann–Whitney Wilcoxon test, while the Kruskal–Wallis test was employed for discrete categorical variables. A *p* value less than 0.05 was considered statistically significant.

## Results

### Distribution and clinicogenomic characteristics of *FGFR1* copy number subgroups in TNBC

One hundred and eighty‐two samples (Table 1) were categorized into four groups based on the FISH results: (1) group 1 (*FGFR1/*CEN8 ratio ≥ 2.0 and mean *FGFR1* signals per cell ≥ 4.0), (2) group 2 (*FGFR1/*CEN8 ratio ≥ 2.0 and mean *FGFR1* signals per cell < 4.0), (3) group 3 (*FGFR1/*CEN8 ratio < 2.0 and mean *FGFR1* signals per cell ≥ 4.0), and (4) group 4 (*FGFR1/*CEN8 ratio < 2.0 and mean *FGFR1* signals per cell < 4.0). The distribution of the samples was as follows: group 1 comprised 8.2% (15/182) of the cohort; group 2 comprised 2.7% (5/182); group 3 comprised 17.6% (32/182); and group 4 comprised 71.4% (130/182) (Figure 1A,B). No significant differences were observed in the clinicopathological characteristics of TNBC patients with different copy number subgroups, except for Ki‐67 indices. Ki‐67 indices were significantly higher in group 1 and group 3 tumours compared with group 4 tumours (*p* = 0.047 and 0.038, respectively), while these differences were no longer statistically significant after adjustment (Bonferroni correction, adjusted *p* = 0.281 and 0.226, respectively) (supplementary material, Table S6). Notably, no statistically significant associations were observed between the *FGFR1* copy number‐based subgroups and the conventional molecular subtypes of TNBC (Table 1; supplementary material, Figure S1). We next analysed genomic features across the four *FGFR1* copy number subgroups in 83 tumours. Oncoplot analysis unveiled single‐nucleotide and copy number variation patterns (Figure 1C). The most prominent cancer‐related variations observed in this cohort were *TP53* mutations (found in 73.5% of tumours), followed by *KMT2C* (28.9%), *MUC16* (21.7%), and *PIK3CA* (19.3%) mutations. The top three somatic copy number variations (including amplification and deletion) with the highest frequency in patients affected the genes *TOP2A* (56.6%), *CD274* (42.2%), and *AURKA* (33.7%), respectively. Notably, *FGFR1* exhibited a 14.5% frequency of copy number alterations (amplification, 9.6%), and a positive correlation was observed between *FGFR1* copy numbers determined by FISH and NGS (supplementary material, Figure S2). While no differences in ITH and MSAF were observed among the subgroups, TMB exhibited an upward trend in group 3 (*p* = 0.02) and group 4 (*p* = 0.048) when compared with group 1 (Figure 1D). Subsequent evaluation of genomic signatures demonstrated no statistically significant distribution differences among the subgroups (Figure 1E).

**Table 1:** Baseline clinicopathological characteristics of triple‐negative breast cancer samples analysed by *FGFR1* FISH and Master Panel sequencing.

Variables, *n* (%)	*FGFR1* FISH (*n =* 182)	Master Panel sequencing (*n =* 88)
*FGFR1*		
Group 1	15 (8.2%)	15 (17.0%)
Group 2	5 (2.7%)	5 (5.7%)
Group 3	32 (17.6%)	28 (31.8%)
Group 4	130 (71.4%)	40 (45.5%)
HER2		
Low	84 (46.2%)	35 (39.8%)
Zero	98 (53.8%)	53 (60.2%)
Histology		
IBC‐NST	172 (94.5%)	85 (96.6%)
Other	10 (5.5%)	3 (3.4%)
Histological grade		
Grade 1	4 (2.2%)	1 (1.1%)
Grade 2	39 (21.4%)	15 (17.0%)
Grade 3	133 (73.1%)	70 (79.5%)
Unknown	6 (3.3%)	2 (2.3%)
Ki‐67 index (%, mean ± SD)	55 ± 27	64 ± 24
Tumour location		
Primary tumour	173 (95.1%)	82 (93.2%)
Recurrent tumour	7 (3.8%)	4 (4.5%)
Lymphatic metastasis	2 (1.1%)	2 (2.3%)
Neoadjuvant therapy		
Treated	9 (4.9%)	5 (5.7%)
Untreated	173 (95.1%)	83 (94.3%)
TNBC subtype (*n* = 166 or 79)*		
IHC‐LAR	55 (33.1%)	18 (22.8%)
IHC‐IM	43 (25.9%)	24 (30.4%)
IHC‐BLIS	47 (25.8%)	26 (32.9%)
IHC‐MES	3 (1.8%)	3 (3.8%)
IHC‐UC	18 (10.8%)	8 (10.1%)

BLIS, basal‐like immune‐suppressed; IBC‐NST, invasive breast carcinoma of no special type; IHC, immunohistochemistry; IM, immunomodulatory; LAR, luminal androgen receptor; MES, mesenchymal; UC, unclassifiable.

* Some samples lacked sufficient tissue for IHC‐based molecular subtyping.

### Differential enrichment of biological process among *FGFR1* copy number subgroups

Eighty‐eight samples met quality criteria for comprehensive genomic profiling and targeted RNA sequencing (Table 1). Differential expression gene (DEG) analysis across the four *FGFR1* copy number subgroups revealed divergent trends in tumour cell proliferation and invasion and immune‐activating molecules. The expression of genes related to tumour cell proliferation and invasion was found to be upregulated, such as *AREG* and *GAB2* in group 1, and *CEACAM6* and *PI3K3CA* in group 2. In addition, group 4 displayed significant upregulation of *HLA‐DQB2*, *IGLL1*, and *CXXC4*. In contrast, group 1 showed downregulation of *CXCL13* and *IFI27*; group 2 exhibited reduced expression of *HLA‐DRB4*, *HLA‐DQA2*, and *IGLL1*. Group 3 demonstrated downregulation of *HLA‐G*, *HLA‐DQB1*, and *HLA‐DQB2* (Figure 2A). Functional annotation of these DEGs was performed using GO, KEGG, Hallmark, WikiPathways, and Reactome analyses. Group 4 was predominantly enriched in immune activation pathways, such as leukocyte‐mediated immunity and T‐cell activation. Conversely, epithelial cell proliferation regulation, Notch signalling, and DNA repair pathways were enriched in group 1. Group 2 displayed pronounced enrichment of the PI3K–Akt, HIF‐1, and JAK–STAT signalling pathways, while group 3 showed particularly active cytokine networks, including cytokine–cytokine receptor interactions and the Ras signalling pathway **(**Figure 2B).

**Figure 2:**
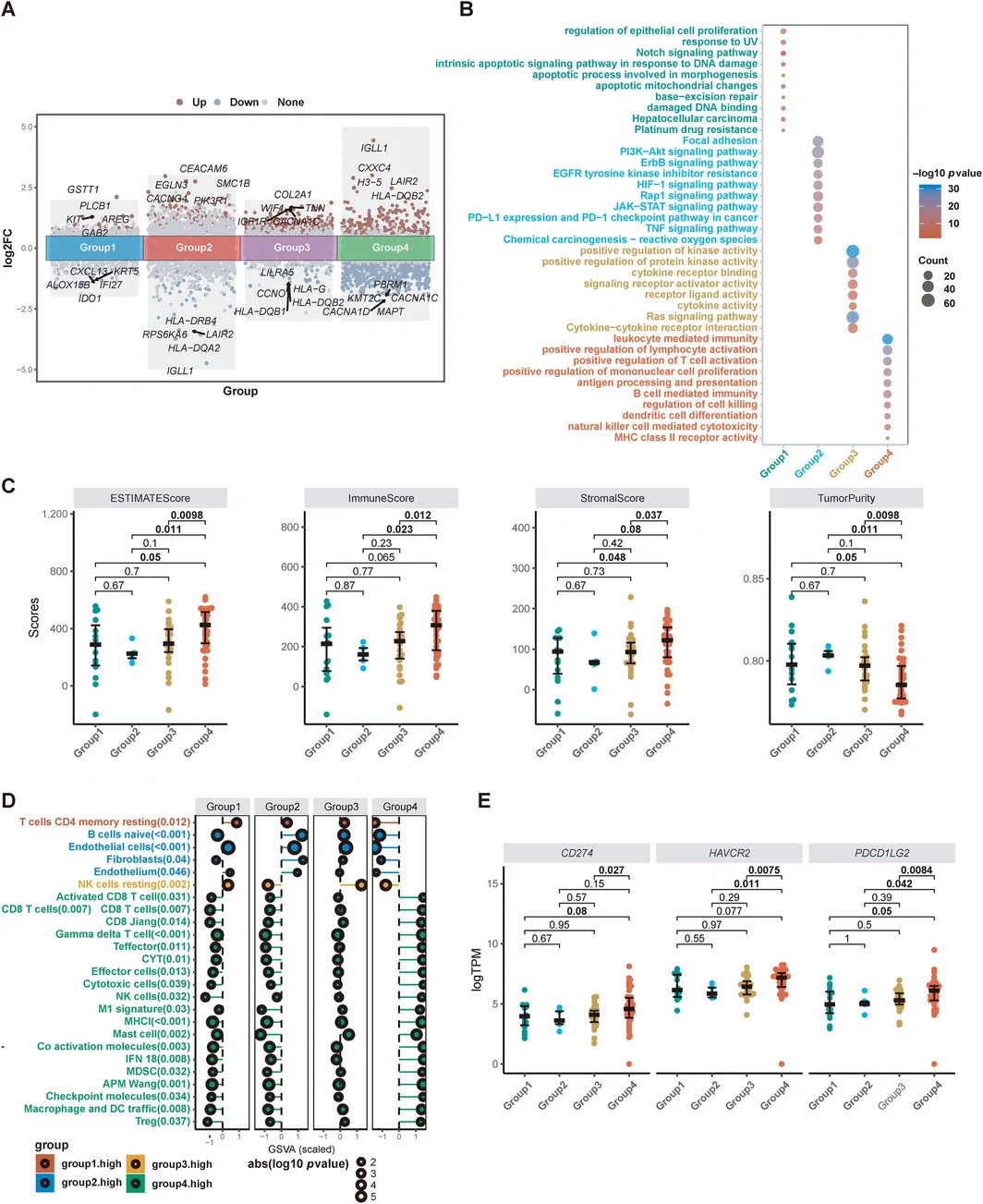
Tumour microenvironment differences in *FGFR1* copy number subgroups. (A) Differential gene expression and (B) pathway enrichment analysis across *FGFR1* status groups. (C) The analysis utilizes the ESTIMATE algorithm to quantify the immune and stromal scores, tumour purity, and overall ESTIMATE scores for each *FGFR1* status group. (D) Immune landscape and GSVA analysis across different *FGFR1* status groups. (E) Expression levels of immune checkpoint molecules across different *FGFR1* status groups. *p* values of statistical significance are shown in bold.

### TME differences among *FGFR1* copy number subgroups

We analysed RNA sequencing results to explore the immune microenvironment characteristics among the four *FGFR1* copy number subgroups. The ESTIMATE analysis revealed a trend of increased stromal and immune scores, alongside decreased tumour purity in samples of group 4 compared with the other three groups (Figure 2C). Group 4 demonstrated significant infiltration of activated immune components, including cytotoxic CD8 T cells, NK cells, and effector populations, along with elevated immune activation signatures. Conversely, groups 1 and 2 had reduced immune cell recruitment and a suppressive microenvironment. Group 3 had a mixed immune profile, intermediate between the active state of group 4 and the depleted state of groups 1 and 2 (Figure 2D). Notably, groups 1 and 2 exhibited significant downregulation of activated immune populations (including activated CD8 T cells, M1 macrophages, and NK cells), whereas group 3 demonstrated selective enrichment of NK cells without comparable increases in other effector subsets (Figure 2D). Immune checkpoint gene expression analysis revealed that group 4 demonstrated elevated levels of *CD274*, *HAVCR2*, and *PDCD1LG2*, compared with the other three groups (Figure 2E). In terms of immunohistochemistry (IHC) staining intensity and cell density values, there was a tendency for more CD3^+^/CD4^+^/CD8^+^ T cells to infiltrate in group 4 (Figure 3A,B).

**Figure 3:**
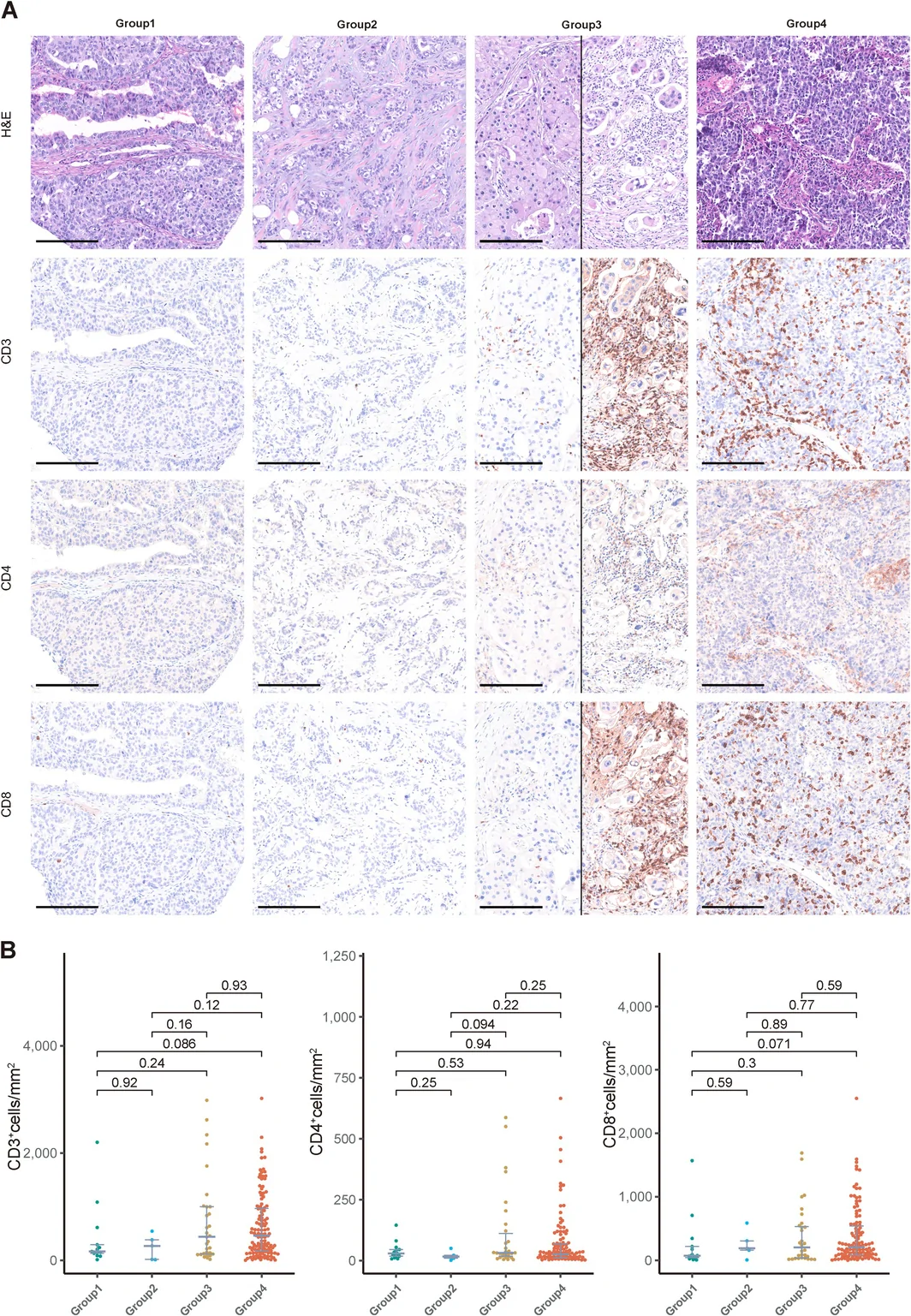
Immunohistochemical validation analysis in *FGFR1* copy number subgroups. (A) Representative IHC staining for CD3^+^/CD4^+^/CD8^+^ cells in the tumour immune microenvironment of each *FGFR1* status group. To clearly illustrate the heterogeneity among the samples in group 3 (with different *FGFR1* gene copy number states, CN ≥ 5 versus CN < 5), we present two representative cases separately. Scale bars: 200 μm. (B) Quantification of IHC positive CD3^+^/CD4^+^/CD8 cells across the *FGFR1* groups.

### *FGFR1* copy number in *FGFR1* group 3 impacts TME

At the genomic level, group 3 exhibits similarities to group 4 and is characterized by an elevated tumour mutational burden. Conversely, within the context of the tumour microenvironment (TME), the associated immune activation features in group 3 are neither downregulated, as observed in groups 1 and 2, nor upregulated, as seen in group 4. Regarding the heterogeneous nature of *FGFR1* copy numbers in group 3, it is worthwhile investigating copy number‐dependent biological behaviour together with TME properties. TNBC samples in group 3 were further stratified based on CN < 5 and CN ≥5 of the *FGFR1* average copy number (CN) [12, 14]. In group 3, patients with *FGFR1* CN ≥ 5 exhibited significant enrichment of immune‐suppressive genes (e.g. *IL6ST*, *FAS*, *CTNNA3*, and *DTX1*), along with activation of pathways related to MAPK signalling, epithelial cell proliferation, and neutrophil migration. Their TME was predominantly characterized by peripheral T cells and resting mast cells. Conversely, patients with *FGFR1* CN < 5 showed enrichment of immune‐activating genes (e.g. *IL11*) and pathways associated with immune activation, interferon‐γ response, T‐cell differentiation, and B‐cell activation. Their TME displayed higher infiltration of cytotoxic CD8 T cells and NK cells (Figure 4A–C).

**Figure 4:**
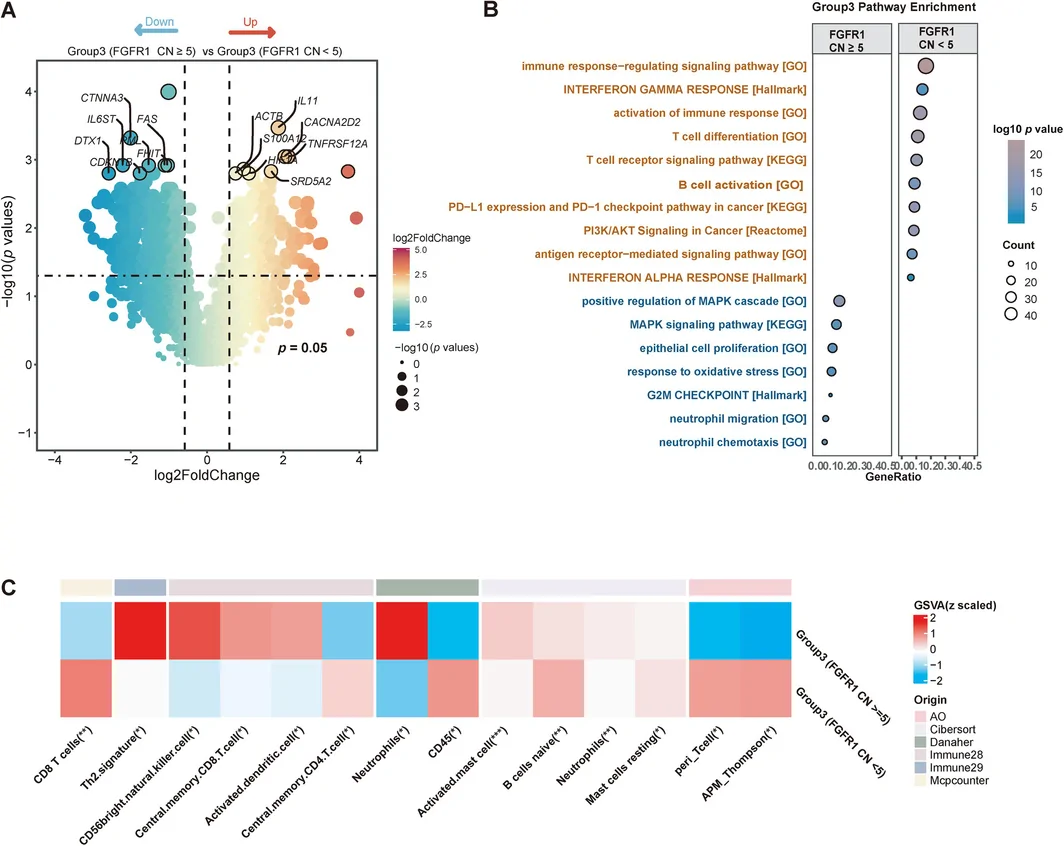
Variations in the tumour microenvironment across the group 3 subtype. (A) Volcano plot showing differentially expressed genes in group 3 (CN ≥ 5) compared with group 3 (CN < 5) patients (fold‐change > 1.5, *p* < 0.05). (B) Enrichment analysis of KEGG, Hallmark, Reactome, and GO terms of the DEGs upregulated in the group 3 (CN ≥ 5) and group 3 (CN < 5) groups. (C) Heatmap illustrating the normalized gene set variation analysis (GSVA) enrichment scores of various tumour microenvironment signatures across the group 3 (CN ≥ 5) and group 3 (CN < 5) groups.

### Proposed criteria of *FGFR1* amplification in TNBC

In group 3, patients were stratified based on *FGFR1* CN, where a CN cutoff ≥ 5 identified a subset with molecular features resembling groups 1 and 2, characterized by enhanced pro‐oncogenic signalling pathway activation. For this study, tumours with *FGFR1* amplified (*FGFR1‐*Amp) were defined as those meeting either of the following criteria: *FGFR1/*CEN8 ≥ 2.0 or CN ≥ 5.0. Tumours not fulfilling these thresholds were classified as *FGFR1*‐neutral (*FGFR1*‐Neu). Based on these criteria, we identified 26 tumours (26/182, 14.29%) with *FGFR1* amplification (*FGFR1‐*Amp) and 156 cases without *FGFR1* amplification (*FGFR1*‐Neu). There were no significant differences in the clinicopathological characteristics between the two groups (Table 2). Differential gene expression analysis revealed that *FGFR1*‐Amp tumours were enriched in tumour proliferation pathways, with significant upregulation of *FGFR1* pathway molecules such as *AKT1/2*, *ARAF*, *MTOR*, *PIK3CA*, and *FGF12/13* (Figure 5A,B). In contrast, *FGFR1*‐Neu tumours exhibited enrichment in immune activation pathways and higher immune cell infiltration (Figure 5A–C). Notably, IHC staining confirmed these findings, showing greater CD3^+^/CD8^+^ T‐cell infiltration in *FGFR1*‐Neu tumours compared with *FGFR1*‐Amp tumours (Figure 5D).

**Table 2:** Comparison of the clinicopathological features in TNBCs with amplified or neutral *FGFR1* FISH results. Data are presented as number (percentage) or median (range). Amplification is defined as *FGFR1/*CEN8 ratio ≥ 2.0 or *FGFR1* copy number ≥ 5.0, while other cases are neutral.

	*FGFR1 FISH*			*P1*	*P2*
	Amplified (*n* = 24)	Neutral (*n* = 154)	Neutral with MP results (*n* = 61)		
Age (range), years	51 (33–78)	51.5 (26–87)	47 (26–83)	0.828	0.113
Menopause				0.678	0.371
Pre‐ and peri‐	12 (50.0%)	70 (45.5%)	37 (60.7%)		
Post‐	12 (50.0%)	84 (54.5%)	24 (39.3%)		
AJCC stage				0.218	0.096
I	12 (50.0%)	65 (42.2%)	19 (31.1%)		
II	6 (25.0%)	68 (44.2%)	32 (52.5%)		
III	5 (20.8%)	18 (11.7%)	8 (13.1%)		
IV	1 (4.2%)	2 (1.3%)	2 (3.3%)		
Unknown	0 (0)	1 (0.6%)	0 (0)		
Histology*				1.000	1.000
IBC‐NST	25 (96.2%)	147 (94.2%)	60 (96.8%)		
Other	1 (3.8%)	9 (5.8%)	2 (3.2%)		
Histological grade*				0.189	0.047
Grade 1	1 (3.8%)	3 (1.9%)	0 (0)		
Grade 2	3 (11.5%)	36 (23.1%)	12 (19.4%)		
Grade 3	20 (76.9%)	113 (72.4%)	50 (80.6%)		
Unknown	2 (7.7%)	4 (2.6%)	0 (0)		
HER2 expression*				0.395	0.871
Low	10 (38.5%)	74 (47.4%)	25 (40.3%)		
Zero	16 (61.5%)	82 (52.6%)	37 (59.7%)		
Ki‐67 index (range), %*	70 (10–95)	60 (5–90)	70 (8–90)	0.050	0.757
Death				1.000	0.928
Present	2 (8.3%)	12 (7.8%)	3 (4.9%)		
Absent	22 (91.7%)	142 (92.2%)	58 (95.1%)		
Other malignancies				0.237	0.415
Present	1 (4.2%)	24 (15.6%)	8 (13.1%)		
Absent	23 (95.8%)	130 (84.4%)	53 (86.9%)		
Family history of malignancies				0.390	0.585
Breast cancer	4 (16.7%)	14 (9.1%)	6 (9.8%)		
Other malignancies	4 (16.7%)	22 (14.3%)	9 (14.8%)		
Absent	16 (66.7%)	118 (76.6%)	46 (75.4%)		

*P1*: the *p* value between *FGFR1*‐amplified and *FGFR1*‐neutral cases; *P2*: the *p* value between *FGFR1*‐amplified cases and *FGFR1*‐neutral cases with MP results.

FISH, fluorescence *in situ* hybridization; MP, Master Panel; TNBC, triple‐negative breast cancer.

* The majority of clinical data were analysed at the patient level, whereas tumour‐specific characteristics, including histological type, tumour grade, HER2 status, and Ki‐67 index, were assessed on a per‐tumour basis.

**Figure 5:**
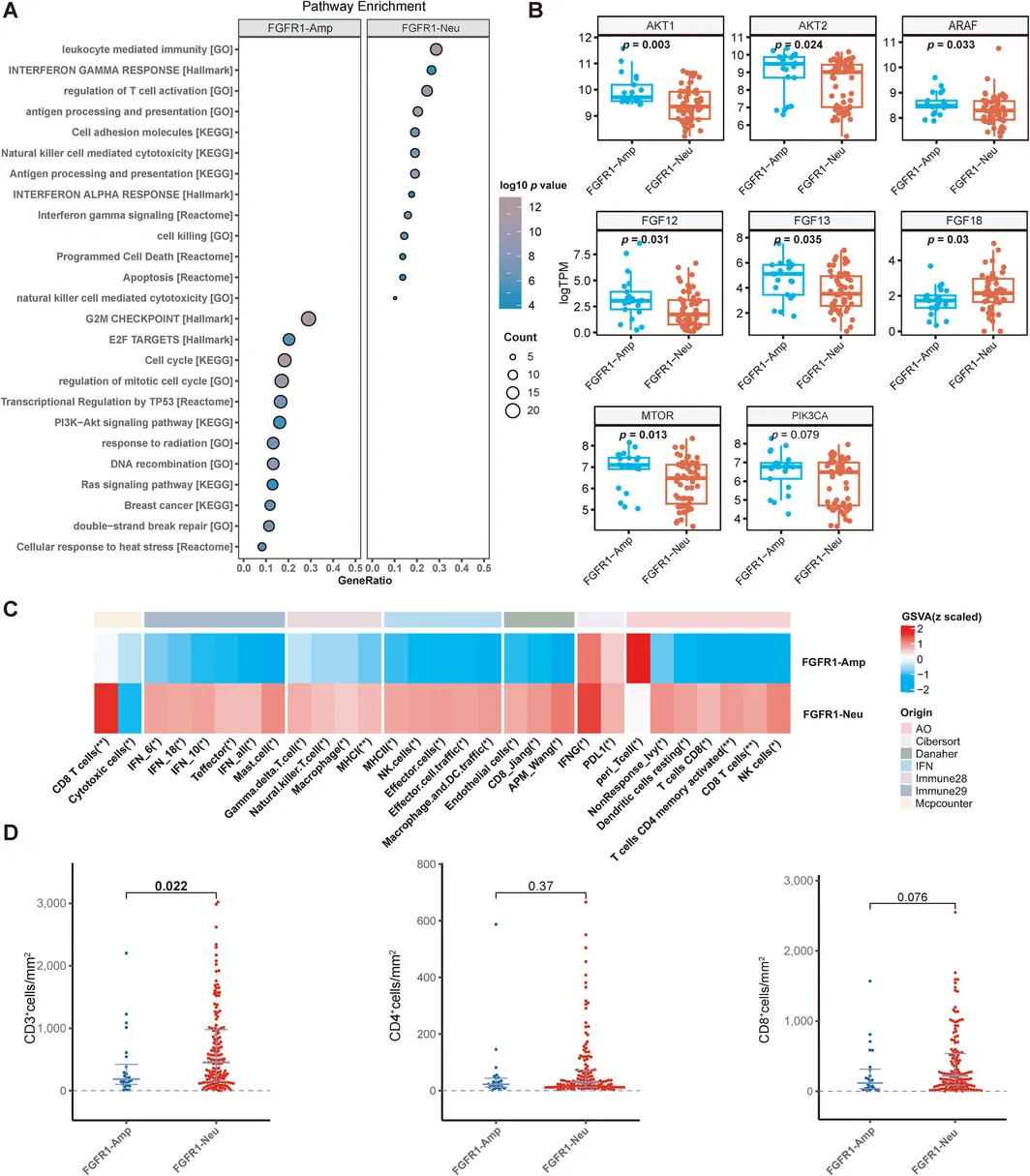
*FGFR1* amplified and its impact on the tumour microenvironment. (A) Enrichment analysis of KEGG, Hallmark, Reactome, and GO terms of the DEGs upregulated in the *FGFR1*‐Amp and *FGFR1*‐Neu groups. (B) Differentially expressed genes in the *FGFR1* signalling pathway between the *FGFR1‐*Amp and *FGFR1*‐Neu groups. (C) Heatmap illustrating the normalized gene set variation analysis (GSVA) enrichment scores of various tumour microenvironment signatures across the *FGFR1*‐Amp and *FGFR1*‐Neu groups. (D) Quantification of IHC positive CD3^+^/CD4^+^/CD8 cells in the *FGFR1*‐Amp and *FGFR1*‐Neu groups. *p < 0.05, **p < 0.01, ***p < 0.001. *p* values of statistical significance are shown in bold.

### *FGFR1* and HER2 status synergistically delineated TNBC into biologically distinctive subcategories

Immune profiling revealed that *FGFR1*‐Amp & HER2‐low tumours exhibited the lowest stromal and immune scores and elevated tumour purity in samples (Figure 6A). Simultaneously, *FGFR1*‐Amp & HER2‐low tumours demonstrated increased tumour‐associated signalling activities, including epithelial–mesenchymal transition (EMT) and enhanced expression of endothelium and angiogenesis signatures, in comparison to the other subgroups (Figure 6B). Conversely, *FGFR1*‐Neu tumours, particularly those with HER2‐zero, showed increased immune cell infiltration. This involved NK cells, effector cells, MHC I/II molecules, and various other immune activation markers (Figure 6B). Remarkably, immune checkpoint markers (*PDL1*, *CTLA4*, *HAVCR2*, *LAG3*, *PDCD1*, and *PDCD1LG2*) were most prominently elevated in *FGFR1*‐Neu & HER2‐low/zero compared with *FGFR1‐*Amp & HER2‐low tumours (Figure 6C). GSEA analysis revealed that *FGFR1*‐Amp tumours were significantly enriched for DNA repair, kinase activity, and cell cycle pathways; TP53 binding was notably enriched in tumours characterized by both *FGFR1* amplification and low HER2 expression (Figure 6D), while antigen processing pathways were significantly enriched in the *FGFR1*‐Neu tumours, particularly those with HER2‐zero.

**Figure 6:**
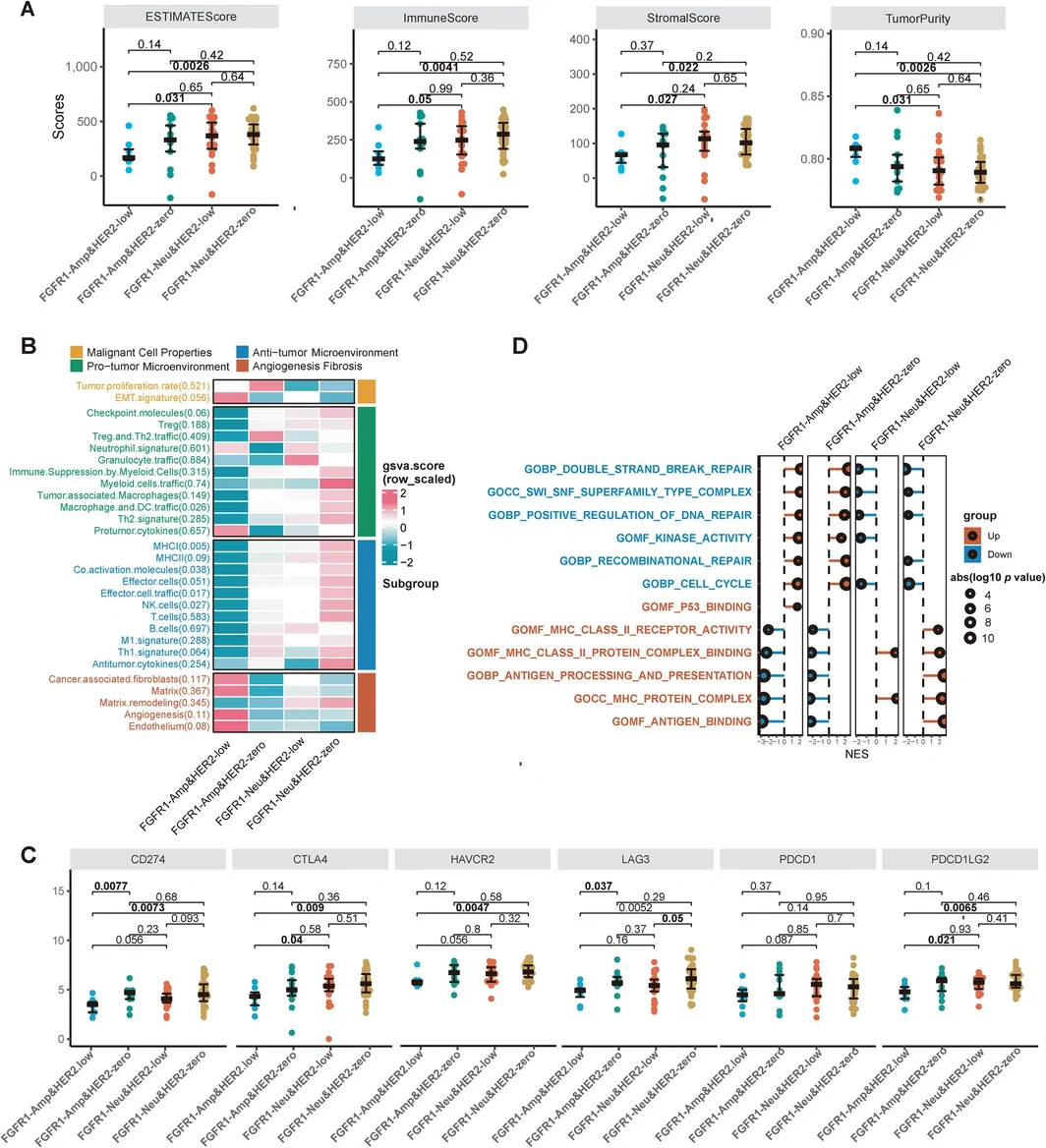
Immune profiles stratified by *FGFR1* and HER2 status. (A) The analysis utilizes the ESTIMATE algorithm to quantify the immune and stromal scores, tumour purity, and overall ESTIMATE scores for each subtype. (B) Immune landscape and GSVA analysis across four distinct subtypes. (C) Expression levels of immune checkpoint molecules across the four different groups. (D) GSEA pathway enrichment analysis across the four groups. *p* values of statistical significance are shown in bold.

## Discussion

In this study, the *FGFR1* amplification status was proposed based on the *FGFR1/*CEN8 ratio and *FGFR1* CN. *FGFR1*‐amplified tumours were defined as those with an *FGFR1* ratio ≥ 2.0 (*FGFR1/*CEN8) or CN ≥ 5.0. Tumours that did not meet these criteria were considered *FGFR1*‐neutral. While Jafarian *et al* concluded that *FGFR1* amplification lacked prognostic significance in TNBC [22], the distinct biological features between these two groups were supported by our transcriptomic data. In terms of FISH‐based detection, Mayo Clinic Laboratories defines *FGFR1* as amplified when the *FGFR1/*CEN8 ratio exceeds 2.0 or when an average of six or more *FGFR1* copies per tumour nucleus are observed. Tumours not meeting these criteria are deemed *FGFR1*‐negative [9]. However, other studies have used different thresholds for defining *FGFR1* amplification. Luo *et al* defined *FGFR1* amplification as an *FGFR1* CN > 6 or an *FGFR1/CEP8* ratio > 2.2 [23]. Furthermore, some studies have defined *FGFR1* amplification as more than five signals per nucleus in more than 50% of cancer cells or when large gene copy clusters are present [12]. Previous studies have predominantly characterized *FGFR1* amplification through centromere and absolute copy number metrics. Our research extends this framework by utilizing a multi‐omics methodology, which integrates DNA, transcriptomic, and IHC data, to delineate four distinct patient cohorts based on centromere and absolute CN status. Importantly, within the heterogeneous group 3, we further differentiated between oncogenic signalling activation and immune activation characteristics using an *FGFR1* CN threshold of ≥ 5 or < 5. This integrative approach enabled us to establish a more precise molecular definition of *FGFR1* amplification, thereby providing enhanced insights into the molecular mechanisms underpinning this genetic alteration. This advancement holds the potential to significantly enhance the precision of targeted therapies and improve prognosis prediction. Furthermore, the present study provides a comprehensive analysis of the impact of *FGFR1* amplification on the tumour biology and immune microenvironment in TNBC. One of the most striking findings of our study is the differential immune microenvironment observed between *FGFR1*‐Amp and *FGFR1*‐Neu tumours. Our study demonstrated that *FGFR1*‐Neu tumours exhibited enhanced immune cell infiltration, characterized by elevated immune scores, increased immune cell invasion, and upregulation of immune checkpoint markers (*CD274*, *HAVCR2*, *PDCD1LG2*). This immune‐activated phenotype is consistent with previous findings that TNBC subtypes lacking *FGFR1* amplification are more likely to exhibit a robust immune response [8, 24]. The elevated expression of CD3, CD4, and CD8 in *FGFR1*‐Neu groups further supports the notion that these tumours may be more responsive to immunotherapy.

In contrast, *FGFR1*‐Amp tumours were associated with a suppressive immune microenvironment and pro‐tumourigenic signatures. These findings are consistent with previous studies that have implicated *FGFR1* amplification in promoting tumour growth and survival through activation of downstream signalling pathways such as MAPK and PI3K/AKT [2, 25]. Beyond its role in proliferative signalling, FGFR pathway aberrations have also been implicated in chemoresistance across breast cancer subtypes, including TNBC [26]. Given the association of *FGFR1* amplification with an immunosuppressive TME, targeting this pathway represents a rational therapeutic strategy. FGFR inhibitors have been approved for specific solid tumours in recent years, but those targeting TNBC remain experimental. AZD4547 and BLU9931 exhibited efficacy in TNBC PDX/PDO models with enhanced FGFR expression [27]. Compound 6 suppresses TNBC migration and invasion by downregulating pFGFR1 and modulating EMT markers [28]. Clinically, André *et al* reported that dovitinib, a multi‐targeted FGFR inhibitor, showed antitumour activity in *FGFR*‐amplified breast cancer cell lines and may have activity in breast cancers with FGF pathway amplification [5]. Mechanistically, FGFR signalling reshapes the tumour microenvironment to facilitate immune escape [29]. Wu *et al* showed that FGFR blockade disrupts cancer‐associated fibroblast (CAF) function, reduces VCAM‐1‐mediated physical barriers, and enhances CD8^+^ T‐cell infiltration in TNBC [3]. Ruan *et al* further summarized that FGFR signalling upregulates immunosuppressive cytokines (e.g. TGF‐β) and angiogenic factors (e.g. VEGF), making FGFR inhibition a promising strategy to reverse TME immunosuppression in TNBC [8].

Targeting *FGFR1* signalling pathways, either through FGFR inhibitors or combination therapies that include immune modulators, may be a viable approach for these patients [30, 31]. While immune checkpoint blockade (ICB) monotherapy has shown controversial efficacy in *FGFR*‐altered tumours, emerging preclinical and clinical evidence supports the superior antitumour activity of combining FGFR tyrosine kinase inhibitors (FGFR‐TKIs) with ICB compared with either therapy alone [32, 33]. For instance, lenvatinib, a multi‐targeted TKI, synergizes with anti‐PD‐1 therapy by remodelling the immunosuppressive tumour microenvironment, promoting tumour vascular normalization, and amplifying cytotoxic T‐cell activity [34]. This combinatorial approach not only enhances therapeutic efficacy but also induces durable immune memory, as demonstrated in hepatocellular carcinoma models and clinical trials [32]. Such synergy highlights the potential of dual targeting of *FGFR* signalling and immune checkpoints to overcome resistance mechanisms in *FGFR*‐driven malignancies.

Prior research has indicated that HER2*‐*low tumours may possess distinct biological characteristics compared with HER2‐zero tumours [35, 36]. Single‐cell RNA‐sequencing analysis showed that HER2‐low TNBC is more metabolically active and aggressive, whereas HER2‐zero TNBC also demonstrated a more active immune microenvironment, with enhanced macrophage polarization, abundant CD8^+^ T cells, diverse T‐cell receptors, and higher levels of immunotherapy markers, contributing to a better immunotherapeutic response [35]. Our findings underscore a significant interaction between *FGFR1* and HER2 in TNBC. Specifically, tumours exhibiting both *FGFR1* amplification and *HER2* expression displayed the most immunosuppressive phenotype. *FGFR1*‐Amp & HER2‐low tumours showed heightened tumour signalling, including EMT and increased endothelium and angiogenesis markers. Considering the substantial advancements in HER2‐targeted antibody–drug conjugates (ADCs) for breast cancer treatment, especially their notable clinical efficacy in populations with low HER2 expression [37], the therapeutic strategy of combining agents targeting FGFR1 inhibitors with ADCs may constitute a novel and promising avenue for research in patients with *FGFR1*‐Amp/ HER2‐low TNBC. In contrast, *FGFR1*‐Neu tumours, especially HER2‐zero, exhibited greater immune cell infiltration, featuring NK cells, effector cells, MHC I/II molecules, and other immune activation indicators. This immunologically ‘hot’ tumour microenvironment suggests that this particular subgroup may derive greater benefit from immunotherapy [38]. Collectively, these findings provide a useful way to group TNBC patients based on *FGFR1* CN and HER2 levels. This grouping suggests that patients with *FGFR1*‐Neu/HER2‐zero tumours may benefit more from immunotherapy, while those with *FGFR1*‐Amp/HER2‐low tumours could be candidates for treatments that combine FGFR inhibitors with other agents.

This study has several limitations that should be acknowledged. First, the retrospective design and the relatively small sample size of *FGFR1*‐Amp tumours may limit the generalizability of our findings. While we observed significant differences in immune activation between *FGFR1* subtypes, these results need to be validated in larger, prospective studies. Second, although our study provides comprehensive transcriptomic and immune profiling data, the causal relationship between *FGFR1* amplification and immune suppression remains to be established. Functional validation through *in vitro* and *in vivo* models is essential to confirm whether *FGFR1* amplification directly drives immune evasion. Third, the lack of robust follow‐up data prevented a definitive analysis of the prognostic value of *FGFR1* amplification. A prospectively monitored cohort is needed for future validation. Finally, given that the *FGFR1* FISH results were based on TMA samples, cases with signals near the amplification cutoff should be interpreted with caution. In clinical practice, analysis on whole‐tissue sections may be preferable to mitigate potential bias from intratumoural heterogeneity.

## Author contributions statement

YG conceived experiments, collected samples and analysed data. FY analysed data. JP and TS carried out experiments. YG, FY, CZ, SW and ZL interpreted the data. YG drafted the manuscript. All authors have read and approved the final paper.

## Supporting information


**Figure S1.** TNBC subtype distribution across the *FGFR1* copy number subgroups
**Figure S2.** Correlation between *FGFR1* copy numbers (CN) detected by fluorescence *in situ* hybridization (FISH) and next‐generation sequencing (NGS) across the *FGFR1* subgroups
**Table S1.** Immunohistochemical conditions and antibodies used
**Table S2.** List of genes detected for single nucleotide variant (SNV) and insertion/deletion (Indel) analysis in the AmoyDx® Master Panel
**Table S3.** List of genes detected for copy number variation (CNV) analysis in the AmoyDx® Master Panel
**Table S4.** List of genes analysed for fusion and alternative splicing events in the AmoyDx® Master Panel
**Table S5.** List of genes assessed for RNA expression in the AmoyDx® Master Panel
**Table S6.** Clinicopathological characteristics of TNBC patients with different copy number subgroups (*n* = 178)

## Data Availability

The data generated in this study are available within the article and its supplementary data files. Further supporting data are available from the corresponding author upon reasonable request.
