# Supplementary material for: Differential FGFR1 copy number status displays unique tumour immune microenvironment in triple‐negative breast cancer
Source: J Pathol. 2026 Sep 9;270(4):507–20. doi: 10.1002/path.70121 (PMC13728813; doi:10.1002/path.70121)
Supplement: Supplementary file 1 [file PATH-270-507-s001.docx]

**Differential *FGFR1* copy number status displays unique tumour immune microenvironment in triple-negative breast cancer**

Y Gao *et al. J Pathol* <https://doi.org/10.1002/path.70121>

**Supplementary Figures S1 and S2**

**Supplementary Tables S1–S6**

**Figure S1. TNBC subtype distribution across the *FGFR1* copy number subgroups.** (A) Representative immunohistochemical (IHC) staining images of AR, CD8, FOXC1, and DCLK1 in TNBC subtypes. Scale bars, 200 μm. (B) Bar chart showing the percentage distribution of TNBC subtypes (BLIS, IM, LAR, MES, UC) among *FGFR1* groups 1, 2, 3, and 4. LAR, luminal androgen receptor; IM, immunomodulatory; BLIS, basal‐like immune‐suppressed; MES, mesenchymal; UC, unclassifiable.

**Figure S2. Correlation between *FGFR1* copy numbers (CN) detected by fluorescence *in situ* hybridization (FISH) and next-generation sequencing (NGS) across the *FGFR1* subgroups.**

**Table S1.** Immunohistochemical conditions and antibodies used.

| **Antibody** | **Vendor** | **Clone** | **Dilution** | **Antigen retrieval** | **Incubation conditions** | **Assessment** |
| --- | --- | --- | --- | --- | --- | --- |
| CD3* | Leica Biosystems, Cat. # PA0553 | LN10 | NA | ER2 | 15 min, RT | M, C |
| CD4* | Leica Biosystems, Cat. # PA0427 | 4B12 | NA | ER2 | 15 min, RT | M, C |
| CD8* | Leica Biosystems, Cat. # PA0183 | 4B11 | NA | ER2 | 15 min, RT | M, C |
| AR* | ZSGB-BIO, Cat. # ZA-0554 | EP120 | NA | FLEX | 40 min, RT | N |
| FOXC1 | Abcam, Cat. # ab227977 | EPR20685 | 1:100 | ER2 | 25 min, RT | N |
| DCLK1 | Abcam, Cat. # ab109029 | EPR6085 | 1:100 | ER2 | 25 min, RT | C |

*Antibodies against CD3, CD4, CD8, and AR were ready-to-use antibodies without dilution during the staining procedure.

Vendors: Leica Biosystems, Richmond, IL, USA; ZSGB-BIO (Beijing Zhongshan Golden Bridge Biotechnology), Beijing, PR China; Abcam, Cambridge, UK.

NA, not applicable; C, cytoplasmic; M, membrane; N, nuclear; RT, room temperature; ER2, BOND epitope retrieval solution 2; FLEX, Envision FLEX target retrieval solution high pH.

**Table S2.** List of genes detected for single nucleotide variant (SNV) and insertion/deletion (Indel) analysis in the AmoyDx^®^ Master Panel. This table lists the 571 genes included in the AmoyDx^®^ Master Panel for DNA-based detection of SNVs and Indels. The comprehensive gene panel enables the identification of key mutations associated with oncogenic pathways, facilitating an in-depth analysis of genomic aberrations in cancer.

| *ABCB1* | *ABL1* | *ABL2* | *ABRAXAS1* | *ACVR1B* | *AGO1* | *AKT1* | *AKT2* | *AKT3* | *ALK* |
| --- | --- | --- | --- | --- | --- | --- | --- | --- | --- |
| *ALOX12B* | *AMER1* | *AP3B1* | *APC* | *APC2* | *APEX1* | *AR* | *ARAF* | *ARFRP1* | *ARID1A* |
| *ARID1B* | *ARID2* | *ARID5B* | *ASXL1* | *ATM* | *ATR* | *ATRX* | *AURKA* | *AURKB* | *AUTS2* |
| *AXIN1* | *AXIN2* | *AXL* | *B2M* | *BAP1* | *BARD1* | *BCL2* | *BCL2L1* | *BCL2L11* | *BCL2L2* |
| *BCL6* | *BCOR* | *BCORL1* | *BCR* | *BIRC3* | *BLK* | *BLM* | *BMP2* | *BMP4* | *BMPR1A* |
| *BRAF* | *BRCA1* | *BRCA2* | *BRD3* | *BRD4* | *BRIP1* | *BTG1* | *BTK* | *C8orf34* | *CALR* |
| *CARD11* | *CASP8* | *CBFB* | *CBL* | *CBLB* | *CCN6* | *CCND1* | *CCND2* | *CCND3* | *CCNE1* |
| *CD274* | *CD34* | *CD44* | *CD74* | *CD79A* | *CD79B* | *CD80* | *CD86* | *CDA* | *CDC73* |
| *CDH1* | *CDK12* | *CDK4* | *CDK6* | *CDK8* | *CDKN1A* | *CDKN1B* | *CDKN2A* | *CDKN2B* | *CDKN2C* |
| *CEBPA* | *CHD2* | *CHD4* | *CHEK1* | *CHEK2* | *CIC* | *CORO2A* | *CREBBP* | *CRKL* | *CRLF2* |
| *CSF1* | *CSF1R* | *CSF3R* | *CTCF* | *CTLA4* | *CTNNA1* | *CTNNB1* | *CUL3* | *CXCL8* | *CYLD* |
| *CYP19A1* | *CYP2C8* | *CYP2D6* | *DAXX* | *DCUN1D1* | *DDR1* | *DDR2* | *DICER1* | *DIS3* | *DKK3* |
| *DNMT1* | *DNMT3A* | *DOT1L* | *DPYD* | *DYNC2H1* | *EED* | *EGFR* | *EIF1AX* | *EIF4A2* | *EMSY* |
| *ENG* | *EP300* | *EPAS1* | *EPCAM* | *EPHA3* | *EPHA5* | *EPHA6* | *EPHA7* | *EPHB1* | *ERBB2* |
| *ERBB3* | *ERBB4* | *ERCC1* | *ERCC2* | *ERCC3* | *ERG* | *ERRFI1* | *ESR1* | *ETS2* | *ETV1* |
| *ETV4* | *ETV5* | *ETV6* | *EWSR1* | *EZH2* | *F2R* | *FANCA* | *FANCC* | *FANCD2* | *FANCE* |
| *FANCF* | *FANCG* | *FANCI* | *FANCL* | *FANCM* | *FAS* | *FAT1* | *FBXW7* | *FCGR2B* | *FGF10* |
| *FGF14* | *FGF19* | *FGF23* | *FGF3* | *FGF4* | *FGF6* | *FGF7* | *FGFR1* | *FGFR2* | *FGFR3* |
| *FGFR4* | *FGR* | *FH* | *FLCN* | *FLT1* | *FLT3* | *FLT4* | *FOXA1* | *FOXL2* | *FOXO1* |
| *FOXP1* | *FRS2* | *FUBP1* | *FUS* | *FYN* | *GABRA6* | *GATA1* | *GATA2* | *GATA3* | *GATA4* |
| *GATA6* | *GEN1* | *GLI1* | *GNA11* | *GNA13* | *GNAQ* | *GNAS* | *GREM1* | *GRIN2A* | *GRM3* |
| *GSK3B* | *GSTP1* | *H1-2* | *H2BC5* | *H3-3A* | *H3-5* | *H3C2* | *HAMP* | *HAVCR2* | *HCK* |
| *HDAC2* | *HEY1* | *HGF* | *HIF1A* | *HLA-A* | *HLA-B* | *HLA-C* | *HNF1A* | *HOXB13* | *HRAS* |
| *HSD3B1* | *HSP90AA1* | *HSPB1* | *ICOS* | *ICOSLG* | *IDH1* | *IDH2* | *IFNGR1* | *IFNGR2* | *IGF1* |
| *IGF1R* | *IGF2* | *IKBKE* | *IKZF1* | *IL13* | *IL1A* | *IL4* | *IL6* | *IL7R* | *INHBA* |
| *INPP4A* | *INPP4B* | *INSR* | *IP6K1* | *IRF1* | *IRF2* | *IRF4* | *IRS2* | *ITGB2* | *ITGB6* |
| *JAK1* | *JAK2* | *JAK3* | *JUN* | *KDM5A* | *KDM5C* | *KDM6A* | *KDR* | *KEAP1* | *KEL* |
| *KIT* | *KLF4* | *KLHL6* | *KMT2A* | *KMT2B* | *KMT2C* | *KMT2D* | *KRAS* | *LAG3* | *LATS1* |
| *LATS2* | *LCK* | *LGALS3* | *LIG4* | *LIN28B* | *LMO1* | *LRP1B* | *LYN* | *LZTR1* | *MAGI2* |
| *MAP2K1* | *MAP2K2* | *MAP2K4* | *MAP3K1* | *MAP3K13* | *MAP3K14* | *MAPK1* | *MAPK3* | *MAPK4* | *MAX* |
| *MCL1* | *MDC1* | *MDM2* | *MDM4* | *MED12* | *MEF2B* | *MEN1* | *MET* | *MGA* | *MGME1* |
| *MGMT* | *MIF* | *MITF* | *MKI67* | *MLH1* | *MLH3* | *MMP1* | *MMP7* | *MPL* | *MPO* |
| *MRE11* | *MSH2* | *MSH3* | *MSH6* | *MST1R* | *MT2A* | *MTHFR* | *MTOR* | *MTRR* | *MUC16* |
| *MUC5B* | *MUTYH* | *MYB* | *MYC* | *MYCL* | *MYCN* | *MYD88* | *MYOD1* | *NAA11* | *NAB2* |
| *NBN* | *NCOA2* | *NCOA3* | *NCOR1* | *NEIL1* | *NF1* | *NF2* | *NFE2L2* | *NFKB1* | *NFKBIA* |
| *NKX2-1* | *NOS2* | *NOS3* | *NOTCH1* | *NOTCH2* | *NOTCH3* | *NOTCH4* | *NPM1* | *NQO1* | *NR1I2* |
| *NR4A3* | *NRAS* | *NRG1* | *NSD1* | *NTRK1* | *NTRK2* | *NTRK3* | *NUP93* | *NUTM1* | *OXSR1* |
| *PAK1* | *PAK3* | *PAK5* | *PALB2* | *PAPPA2* | *PARP1* | *PAX3* | *PAX5* | *PAX7* | *PAX8* |
| *PBRM1* | *PDCD1* | *PDCD1LG2* | *PDGFB* | *PDGFRA* | *PDGFRB* | *PDK1* | *PDPK1* | *PEG3* | *PGR* |
| *PHF6* | *PIK3C2B* | *PIK3C2G* | *PIK3C3* | *PIK3CA* | *PIK3CB* | *PIK3CD* | *PIK3CG* | *PIK3R1* | *PIK3R2* |
| *PIM1* | *PLCG2* | *PLK2* | *PMS1* | *PMS2* | *PNRC1* | *POLD1* | *POLE* | *POLE4* | *PPARG* |
| *PPP2R1A* | *PPP2R2A* | *PRDM1* | *PRDX1* | *PRDX6* | *PREX2* | *PRKAA1* | *PRKACA* | *PRKAR1A* | *PRKCI* |
| *PRKDC* | *PRKN* | *PRSS8* | *PSMD4* | *PTCH1* | *PTEN* | *PTGS2* | *PTPN11* | *PTPRD* | *PTTG1* |
| *PXDNL* | *QKI* | *RAC1* | *RAD21* | *RAD50* | *RAD51* | *RAD51B* | *RAD51C* | *RAD51D* | *RAD52* |
| *RAD54L* | *RAF1* | *RANBP2* | *RARA* | *RASA1* | *RASAL1* | *RB1* | *RBM10* | *RECQL* | *RECQL4* |
| *REL* | *RET* | *REV3L* | *RHEB* | *RHOA* | *RICTOR* | *RIPK4* | *RIT1* | *RNASEL* | *RNF43* |
| *ROBO2* | *ROS1* | *RPPH1* | *RPS6KB1* | *RPS6KB2* | *RPTOR* | *RSF1* | *RUNX1* | *RUNX1T1* | *SCN8A* |
| *SDHA* | *SDHAF2* | *SDHB* | *SDHC* | *SDHD* | *SEMA3C* | *SERPINB3* | *SERPINB4* | *SERPINE1* | *SETBP1* |
| *SETD2* | *SF3B1* | *SIK1* | *SKP2* | *SLC28A3* | *SLC47A1* | *SLCO1B1* | *SLIT2* | *SLX4* | *SMAD2* |
| *SMAD3* | *SMAD4* | *SMARCA4* | *SMARCB1* | *SMARCD1* | *SMO* | *SNCAIP* | *SOCS1* | *SOD2* | *SOX10* |
| *SOX17* | *SOX2* | *SOX9* | *SPEN* | *SPOP* | *SPTA1* | *SRC* | *SRSF2* | *SS18* | *STAG2* |
| *STAT3* | *STAT4* | *STAT6* | *STK11* | *SUFU* | *SUZ12* | *SYK* | *TAF1* | *TAOK1* | *TBX3* |
| *TCF7L1* | *TCF7L2* | *TENT5C* | *TERT* | *TET1* | *TET2* | *TFE3* | *TGFB1* | *TGFBR2* | *TIGIT* |
| *TMEM127* | *TMPRSS2* | *TNF* | *TNFAIP3* | *TNFRSF14* | *TNFRSF18* | *TNFRSF9* | *TOP1* | *TOP2A* | *TP53* |
| *TPMT* | *TRAF3* | *TRAF7* | *TRRAP* | *TSC1* | *TSC2* | *TSHR* | *TXNRD2* | *TYMS* | *U2AF1* |
| *UGT1A1* | *UMPS* | *VEGFA* | *VHL* | *WRN* | *WT1* | *XPC* | *XPO1* | *XRCC1* | *XRCC2* |
| *XRCC3* | *XRCC4* | *XRCC5* | *YES1* | *YWHAE* | *ZBTB2* | *ZFHX4* | *ZNF217* | *ZNF703* | *ZNRF3* |
| *ZRSR2* |  |  |  |  |  |  |  |  |  |

**Table S3.** List of genes detected for copy number variation (CNV) analysis in the AmoyDx^®^ Master Panel. This table contains the 30 genes included in the AmoyDx^®^ Master Panel for CNV analysis. These genes are critical for assessing genomic amplifications and deletions, providing insights into copy number changes that may influence cancer progression and therapeutic response.

| *AKT2* | *AKT3* | *AURKA* | *CCND1* | *CCNE1* | *CD274* | *CDK4* | *CDK6* | *EGFR* | *ERBB2* |
| --- | --- | --- | --- | --- | --- | --- | --- | --- | --- |
| *FGF19* | *FGF3* | *FGFR1* | *FGFR2* | *FGFR3* | *HGF* | *IGF1R* | *MAPK1* | *MDM2* | *MDM4* |
| *MET* | *MYC* | *NTRK3* | *PDGFRA* | *PGR* | *PIK3CA* | *RET* | *RICTOR* | *SMO* | *TOP2A* |

**Table S4.** List of genes analysed for fusion and alternative splicing events in the AmoyDx^®^ Master Panel. This table lists the 347 genes included in the AmoyDx^®^ Master Panel for co-detection of gene fusions and alternative splicing events using DNA and RNA. These analyses support the detection of structural variants and splicing aberrations that may contribute to oncogenic mechanisms and potential treatment targets.

| *ABR* | *ACADM* | *ADAMTS16* | *AFAP1* | *AFF3* | *AGAP3* | *AGBL4* | *AGGF1* | *AGK* | *AGTRAP* |
| --- | --- | --- | --- | --- | --- | --- | --- | --- | --- |
| *AHCYL1* | *AKAP13* | *AKAP9* | *ALAD* | *ALK* | *AMBRA1* | *AMOT* | *AMOTL2* | *ANO3* | *AP1M1* |
| *AP3B1* | *APIP* | *APOLD1* | *AR* | *ARHGEF2* | *ARMC10* | *ASPSCR1* | *ATF7IP* | *ATG7* | *ATIC* |
| *ATP1B1* | *ATP6V1D* | *BAG4* | *BAIAP2L1* | *BCAN* | *BCL2L11* | *BCL2L14* | *BICC1* | *BRAF* | *BTBD1* |
| *BTF3L4* | *C8orf34* | *CACNA2D1* | *CAPZA2* | *CASP7* | *CAV1* | *CBR4* | *CBX5* | *CCAR2* | *CCDC186* |
| *CCDC6* | *CCDC91* | *CD74* | *CDC27* | *CDK12* | *CEP43* | *CEP72* | *CEP85L* | *CEP89* | *CHTOP* |
| *CIDEA* | *CIT* | *CLCN6* | *CLDN18* | *CLIP1* | *CLTC* | *CNIH4* | *CNTRL* | *COL14A1* | *COL16A1* |
| *CREB5* | *CTNNA3* | *CTRC* | *CTTN* | *CUX1* | *CWH43* | *CYSTM1* | *DCTN1* | *DDX21* | *DGCR2* |
| *DHX15* | *DIAPH1* | *DUSP6* | *DYNC1I2* | *DZANK1* | *EEF1G* | *EGFR* | *EIF1* | *EIF2AK3* | *EIF3L* |
| *EIF4A2* | *ELAVL3* | *EML4* | *EPS15* | *ERBB2* | *ERBB4* | *ERC1* | *ERG* | *ERLIN2* | *ERP44* |
| *ESR1* | *ETV1* | *ETV4* | *ETV5* | *ETV6* | *EWSR1* | *EZR* | *F11* | *F11R* | *FAM114A2* |
| *FAM131B* | *FBXO28* | *FCHSD1* | *FGFR1* | *FGFR1OP2* | *FGFR2* | *FGFR3* | *FGFR4* | *FILIP1* | *FIP1L1* |
| *FKBP15* | *FLI1* | *FN1* | *FOXO1* | *GAB2* | *GCGR* | *GHITM* | *GIT2* | *GKAP1* | *GLOD4* |
| *GOLGA5* | *GOPC* | *GRB10* | *GRIPAP1* | *GTF2I* | *GTF2IRD1* | *GYG1* | *HEY1* | *HIP1* | *HLA-A* |
| *HNRNPA2B1* | *HSF2BP* | *IKZF3* | *INSL4* | *IRF2BP2* | *JAKMIP1* | *KCTD8* | *KDM7A* | *KIAA1217* | *KIAA1549* |
| *KIAA1598* | *KIF5B* | *KIT* | *KLC1* | *KLHL7* | *KLK2* | *LIMA1* | *LMNA* | *LOH12CR1* | *LRRC71* |
| *LSM12* | *LSM14A* | *LUC7L2* | *LZTFL1* | *MAD1L1* | *MAGI3* | *MEF2D* | *MET* | *MGEA5* | *MKRN1* |
| *MLLT10* | *MORC3* | *MPRIP* | *MSN* | *MYB* | *MYH9* | *MYO18A* | *MYO5A* | *MYRIP* | *MZT1* |
| *NAB2* | *NACC2* | *NCOA2* | *NCOA4* | *NFASC* | *NFIB* | *NOL4* | *NOS1AP* | *NOTCH2* | *NPM1* |
| *NR4A3* | *NRAP* | *NRBF2* | *NRG1* | *NRG2* | *NRG3* | *NSD1* | *NTRK1* | *NTRK2* | *NTRK3* |
| *NUB1* | *NUP214* | *OCIAD1* | *OFD1* | *OXR1* | *PAN3* | *PAPD7* | *PAPSS1* | *PAWR* | *PAX8* |
| *PCM1* | *PDE7A* | *PDE9A* | *PDGFB* | *PDGFRA* | *PDGFRB* | *PEAR1* | *PGAP3* | *PHC3* | *PHF12* |
| *PHLDB3* | *PLEKHG6* | *PMEPA1* | *POC1B* | *POF1B* | *PPARG* | *PPFIBP1* | *PPHLN1* | *PPL* | *PPP1R1B* |
| *PPP1R21* | *PPP4R3B* | *PRDX1* | *PRKAR1A* | *PRKAR2A* | *PSMB3* | *PSPH* | *PTGFRN* | *PWWP2A* | *QKI* |
| *RAB3IL1* | *RABGAP1L* | *RAD18* | *RAF1* | *RANBP2* | *RBMS3* | *RBPMS* | *REG4* | *RET* | *RNF130* |
| *RNF213* | *ROCK1* | *ROS1* | *RPL3* | *RTN4RL1* | *SARS* | *SCP2* | *SCYL3* | *SDC4* | *SEC22B* |
| *SEC31A* | *SEC61G* | *SEPT10* | *SEPT14* | *SEPTIN3* | *SHROOM3* | *SHTN1* | *SIL1* | *SLC12A7* | *SLC34A2* |
| *SLC3A2* | *SLC43A2* | *SLMAP* | *SND1* | *SORBS1* | *SPAG17* | *SPECC1L* | *SQSTM1* | *SS18* | *SSBP2* |
| *SSX1* | *SSX2* | *ST7* | *STARD3* | *STAT3* | *STAT6* | *STK26* | *STMN2* | *STRN* | *STRN3* |
| *TACC1* | *TACC2* | *TACC3* | *TANK* | *TATDN1* | *TAX1BP1* | *TBC1D1* | *TBC1D2* | *TBL1XR1* | *TEAD2* |
| *TFE3* | *TFG* | *TLCD2* | *TLE4* | *TLE5* | *TMEFF2* | *TMEM106B* | *TMEM165* | *TMPRSS2* | *TP53* |
| *TP73* | *TPD52L1* | *TPM1* | *TPM3* | *TPM4* | *TPR* | *TRAF1* | *TRAF2* | *TRAK1* | *TRIM24* |
| *TRIM27* | *TRIM33* | *TRIM63* | *TXLNA* | *USP10* | *USP8* | *UST* | *VCAN* | *VCL* | *VIM* |
| *VOPP1* | *VSTM2A* | *WAC* | *WDCP* | *WIPF2* | *WNK2* | *XXYLT1* | *YWHAE* | *ZAN* | *ZC3HAV1* |
| *ZCCHC8* | *ZMYM2* | *ZMYM4* | *ZNF34* | *ZNF485* | *ZNF703* | *ZSCAN30* |  |  |  |

**Table S5.** List of genes assessed for RNA expression in the AmoyDx^®^ Master Panel. This table provides the 2,660 genes analysed for RNA expression in the AmoyDx^®^ Master Panel. These genes represent key pathways and molecular signatures, enabling a comprehensive characterization of transcriptomic changes in the tumour microenvironment and cancer biology.

| *A2M* | *CACNB3* | *COL4A1* | *F2RL1* | *HES1* | *KDR* | *MSRB2* | *PLA2G6* | *RXRB* | *TERT* |
| --- | --- | --- | --- | --- | --- | --- | --- | --- | --- |
| *ABCB1* | *CACNB4* | *COL4A2* | *FAAP24* | *HES5* | *KEAP1* | *MST1R* | *PLAT* | *RXRG* | *TET2* |
| *ABCF1* | *CACNG1* | *COL4A3* | *FABP1* | *HEY1* | *KIAA1217* | *MTF1* | *PLAU* | *RYBP* | *TFDP1* |
| *ABL1* | *CACNG4* | *COL4A4* | *FABP4* | *HEY2* | *KIAA1549* | *MTF2* | *PLAUR* | *S100A12* | *TFE3* |
| *ABR* | *CACNG6* | *COL4A5* | *FADD* | *HEYL* | *KIAA1598* | *MTMR14* | *PLCB1* | *S100A2* | *TFEB* |
| *ABTB2* | *CADPS* | *COL4A6* | *FAM114A2* | *HFM1* | *KIF12* | *MTOR* | *PLCB4* | *S100A4* | *TFG* |
| *ACAD9* | *CALM1* | *COL5A1* | *FAM124B* | *HGD* | *KIF2C* | *MTRR* | *PLCD3* | *S100A7* | *TFRC* |
| *ACADM* | *CALM2* | *COL5A2* | *FAM131B* | *HGF* | *KIF5B* | *MUC1* | *PLCE1* | *S100A8* | *TG* |
| *ACAN* | *CALM3* | *COL6A3* | *FAM13C* | *HHEX* | *KIF7* | *MUC2* | *PLCG1* | *S100A9* | *TGFA* |
| *ACOT12* | *CALML3* | *COL6A6* | *FAM167A* | *HHIP* | *KIR2DL1* | *MUC4* | *PLCG2* | *S100B* | *TGFB1* |
| *ACSF3* | *CALML4* | *COLEC12* | *FAM30A* | *HIF1A* | *KIR2DL2* | *MUTYH* | *PLD1* | *S100P* | *TGFB2* |
| *ACTA2* | *CALML5* | *COMP* | *FANCA* | *HIP1* | *KIR2DL3* | *MX1* | *PLD2* | *SAA1* | *TGFB3* |
| *ACTB* | *CALML6* | *CORO1A* | *FANCB* | *HK1* | *KIR2DS4* | *MXI1* | *PLEKHA5* | *SAMD9* | *TGFBR1* |
| *ACTG1* | *CAMK1* | *COX11* | *FANCC* | *HK2* | *KIR3DL1* | *MYB* | *PLEKHG6* | *SAMHD1* | *TGFBR2* |
| *ACTG2* | *CAMK1D* | *COX4I1* | *FANCD2* | *HLA-A* | *KIR3DL2* | *MYBL2* | *PLK1* | *SAMSN1* | *TH* |
| *ACTL6A* | *CAMK1G* | *COX5B* | *FANCE* | *HLA-B* | *KIR3DL3* | *MYC* | *PLK3* | *SAP130* | *THBD* |
| *ACTL6B* | *CAMK2A* | *COX6A1* | *FANCF* | *HLA-C* | *KIR3DS1* | *MYCN* | *PLOD2* | *SARS* | *THBS1* |
| *ACTR3B* | *CAMK2B* | *COX6B1* | *FANCG* | *HLA-DMA* | *KIT* | *MYCT1* | *PMAIP1* | *SBNO2* | *THBS4* |
| *ACVR1B* | *CAMK2D* | *CPA3* | *FANCL* | *HLA-DMB* | *KITLG* | *MYD88* | *PMCH* | *SCGB2A2* | *THEM4* |
| *ACVR1C* | *CAMK2G* | *CPEB2* | *FAP* | *HLA-DOA* | *KLC1* | *MYH9* | *PMEL* | *SCP2* | *THRA* |
| *ACVR2A* | *CAMK4* | *CPSF7* | *FAS* | *HLA-DOB* | *KLF2* | *MYO18A* | *PMEPA1* | *SCUBE2* | *THRB* |
| *ACY1* | *CAMP* | *CR1* | *FASLG* | *HLA-DPA1* | *KLF4* | *MYO5A* | *PML* | *SCYL3* | *THY1* |
| *ADA* | *CAPN2* | *CR2* | *FAU* | *HLA-DPB1* | *KLHL7* | *MYRIP* | *PMS2* | *SDC1* | *TIAM1* |
| *ADAM12* | *CAPN6* | *CRABP2* | *FBP1* | *HLA-DQA1* | *KLK2* | *MZT1* | *PNKP* | *SDC4* | *TICAM1* |
| *ADAMTS16* | *CAPZA2* | *CREB1* | *FBXO28* | *HLA-DQA2* | *KLK3* | *NAALAD2* | *PNMA1* | *SDHA* | *TICAM2* |
| *ADGRE1* | *CARD11* | *CREB3* | *FBXW7* | *HLA-DQB1* | *KLRB1* | *NAB2* | *PNOC* | *SEC22B* | *TIE1* |
| *ADGRE2* | *CARD9* | *CREB3L1* | *FCAR* | *HLA-DQB2* | *KLRC1* | *NACC2* | *PNPLA5* | *SEC31A* | *TIGIT* |
| *ADGRE5* | *CASP1* | *CREB3L2* | *FCER1A* | *HLA-DRA* | *KLRC2* | *NANOG* | *POC1B* | *SEC61G* | *TIRAP* |
| *ADH1A* | *CASP10* | *CREB3L3* | *FCER1G* | *HLA-DRB1* | *KLRD1* | *NASP* | *POF1B* | *SEL1L3* | *TLCD2* |
| *ADH1B* | *CASP12* | *CREB3L4* | *FCER2* | *HLA-DRB3* | *KLRF1* | *NAT1* | *POLB* | *SELE* | *TLE4* |
| *ADH1C* | *CASP3* | *CREB5* | *FCF1* | *HLA-DRB4* | *KLRG1* | *NAT8L* | *POLD1* | *SELENBP1* | *TLE5* |
| *ADH4* | *CASP7* | *CREBBP* | *FCGR1A* | *HLA-DRB5* | *KLRK1* | *NBN* | *POLD2* | *SELENOK* | *TLK2* |
| *ADH6* | *CASP8* | *CRK* | *FCGR2A* | *HLA-E* | *KMT2C* | *NCAM1* | *POLD4* | *SELL* | *TLR1* |
| *ADM* | *CASP9* | *CRKL* | *FCGR2B* | *HLA-F* | *KMT2D* | *NCF1* | *POLE2* | *SELP* | *TLR10* |
| *ADORA2A* | *CAV1* | *CRLF2* | *FCGR3A* | *HLA-F-AS1* | *KRAS* | *NCF4* | *POLK* | *SELPLG* | *TLR2* |
| *AFAP1* | *CBL* | *CRP* | *FCGR3B* | *HLA-G* | *KREMEN1* | *NCL* | *POLR1B* | *SEMA6A* | *TLR3* |
| *AFDN* | *CBLB* | *CRTAM* | *FCGRT* | *HLF* | *KRT1* | *NCOA1* | *POLR1C* | *SEMG1* | *TLR4* |
| *AFF3* | *CBLC* | *CSF1* | *FCHO1* | *HMBS* | *KRT10* | *NCOA2* | *CD3EAP* | *SENP1* | *TLR5* |
| *AGAP3* | *CBR4* | *CSF1R* | *FCHSD1* | *HMGA1* | *KRT13* | *NCOA3* | *POLR2A* | *SEPT10* | *TLR6* |
| *AGBL4* | *CBX5* | *CSF2* | *FCN1* | *HMGA2* | *KRT14* | *NCOA4* | *POLR2D* | *SEPT14* | *TLR7* |
| *AGGF1* | *CC2D1B* | *CSF2RB* | *FCRL2* | *HMGB1* | *KRT15* | *NCOR1* | *POLR2H* | *SEPTIN3* | *TLR8* |
| *AGK* | *CCAR2* | *CSF3* | *FCRLA* | *HMGN5* | *KRT17* | *NCOR2* | *POLR2J* | *SERINC1* | *TLR9* |
| *AGR2* | *CCDC186* | *CSF3R* | *FEN1* | *HMOX1* | *KRT18* | *NCR1* | *POLR3G* | *SERINC2* | *TLX1* |
| *AGTRAP* | *CCDC198* | *CSNK1A1* | *FEZ1* | *HNF1A* | *KRT19* | *NCR3* | *POSTN* | *SERINC3* | *TM4SF4* |
| *AHCYL1* | *CCDC6* | *CSNK1A1L* | *FGF1* | *HNRNPA2B1* | *KRT20* | *NDC1* | *POU2AF1* | *SERINC5* | *TMEFF2* |
| *AHR* | *CCDC91* | *CST2* | *FGF10* | *HNRNPL* | *KRT5* | *NDC80* | *POU2F2* | *SERPINA1* | *TMEM106B* |
| *AICDA* | *CCL1* | *CT45A1* | *FGF11* | *HOXA10* | *KRT6A* | *NDUFA1* | *POU5F1* | *SERPINA3* | *TMEM140* |
| *AIF1* | *CCL11* | *CTAG1B* | *FGF12* | *HOXA11* | *KRT6B* | *NDUFA11* | *PPA1* | *SERPINB2* | *TMEM163* |
| *AIRE* | *CCL13* | *CTAG2* | *FGF13* | *HOXA9* | *KRT6C* | *NDUFA12* | *PPAN* | *SERPINB3* | *TMEM165* |
| *AKAP1* | *CCL14* | *CTAGE1* | *FGF14* | *HOXC10* | *KRT7* | *NDUFA13* | *PPARD* | *SERPINB5* | *TMEM43* |
| *AKAP13* | *CCL15* | *CTBP1* | *FGF16* | *HOXD11* | *KYAT1* | *NDUFA2* | *PPARG* | *SERPINE1* | *TMEM45B* |
| *AKAP9* | *CCL16* | *CTBP2* | *FGF17* | *HPGD* | *L1CAM* | *NDUFA3* | *PPARGC1A* | *SERPING1* | *TMPRSS2* |
| *AKR1C3* | *CCL17* | *CTCFL* | *FGF18* | *HPRT1* | *LAG3* | *NDUFA4L2* | *PPARGC1B* | *SERPINH1* | *TMPRSS3* |
| *AKR1C4* | *CCL18* | *CTLA4* | *FGF19* | *HRAS* | *LAIR1* | *NDUFA6* | *PPAT* | *SETBP1* | *TMPRSS4* |
| *AKT1* | *CCL19* | *CTNNA1* | *FGF2* | *HSD11B1* | *LAIR2* | *NDUFA7* | *PPBP* | *SETD2* | *TMUB2* |
| *AKT2* | *CCL2* | *CTNNA2* | *FGF20* | *HSD17B8* | *LAMA1* | *NDUFB1* | *PPFIBP1* | *SF3A1* | *TNC* |
| *AKT3* | *CCL20* | *CTNNA3* | *FGF21* | *HSDL2* | *LAMA2* | *NDUFB10* | *PPHLN1* | *SF3A3* | *TNF* |
| *ALAD* | *CCL21* | *CTNNB1* | *FGF22* | *HSF2BP* | *LAMA3* | *NDUFB11* | *PPIA* | *SF3B1* | *TNFAIP3* |
| *ALAS1* | *CCL22* | *CTRC* | *FGF23* | *HSP90AA1* | *LAMA4* | *NDUFB4* | *PPL* | *SFN* | *TNFAIP6* |
| *ALCAM* | *CCL23* | *CTSC* | *FGF3* | *HSP90AB1* | *LAMA5* | *NDUFB7* | *PPP1R1B* | *SFRP1* | *TNFAIP8* |
| *ALDOA* | *CCL24* | *CTSG* | *FGF4* | *HSP90B1* | *LAMB1* | *NDUFB8* | *PPP1R21* | *SFRP2* | *TNFRSF10A* |
| *ALDOC* | *CCL25* | *CTSH* | *FGF5* | *HSPA1A* | *LAMB2* | *NDUFS7* | *PPP2CB* | *SFRP4* | *TNFRSF10B* |
| *ALK* | *CCL26* | *CTSL* | *FGF6* | *HSPA2* | *LAMB3* | *NDUFS8* | *PPP2R1A* | *SFTPB* | *TNFRSF10C* |
| *ALKBH2* | *CCL27* | *CTSS* | *FGF7* | *HSPA6* | *LAMB4* | *NECTIN1* | *PPP2R2B* | *SFTPC* | *TNFRSF10D* |
| *ALKBH3* | *CCL28* | *CTSV* | *FGF8* | *HSPB1* | *LAMC1* | *NECTIN2* | *PPP2R2C* | *SFXN1* | *TNFRSF11A* |
| *ALOX15B* | *CCL3* | *CTSW* | *FGF9* | *HTR3A* | *LAMC2* | *NECTIN4* | *PPP2R3A* | *SGK1* | *TNFRSF11B* |
| *AMBP* | *CCL3L1* | *CTTN* | *FGFR1* | *HYDIN* | *LAMC3* | *NEFL* | *PPP3CA* | *SGK2* | *TNFRSF12A* |
| *AMBRA1* | *CCL4* | *CUL1* | *FGFR1OP2* | *IBSP* | *LAMP1* | *NEIL1* | *PPP3CB* | *SH2B2* | *TNFRSF13B* |
| *AMER1* | *CCL5* | *CUL2* | *FGFR2* | *ICAM1* | *LAMP2* | *NEIL3* | *PPP3CC* | *SH2B3* | *TNFRSF13C* |
| *AMH* | *CCL7* | *CUL3* | *FGFR3* | *ICAM2* | *LAMP3* | *NF1* | *PPP3R1* | *SH2D1A* | *TNFRSF14* |
| *AMMECR1L* | *CCL8* | *CUX1* | *FGFR4* | *ICAM3* | *LAPTM5* | *NF2* | *PPP3R2* | *SH2D1B* | *TNFRSF17* |
| *AMOT* | *CCNA1* | *CWH43* | *FH* | *ICAM4* | *LAT* | *NFAM1* | *PPP4R3B* | *SHC1* | *TNFRSF18* |
| *AMOTL2* | *CCNA2* | *CX3CL1* | *FHIT* | *ICAM5* | *LBP* | *NFASC* | *PRAME* | *SHC2* | *TNFRSF19* |
| *ANGPT1* | *CCNB1* | *CX3CR1* | *FILIP1* | *ICOS* | *LCK* | *NFATC1* | *PRC1* | *SHC3* | *TNFRSF1A* |
| *ANGPT2* | *CCNB2* | *CXADR* | *FIP1L1* | *ICOSLG* | *LCN2* | *NFATC2* | *PRCC* | *SHC4* | *TNFRSF1B* |
| *ANGPTL4* | *CCNB3* | *CXCL1* | *FKBP15* | *ID1* | *LCOR* | *NFATC3* | *PRDM1* | *SHH* | *TNFRSF25* |
| *ANKLE2* | *CCND1* | *CXCL10* | *FLCN* | *ID2* | *LCP1* | *NFATC4* | *PRDM6* | *SHROOM3* | *TNFRSF4* |
| *ANKRD28* | *CCND2* | *CXCL11* | *FLG* | *ID3* | *LDHA* | *NFE2L2* | *PRDX1* | *SHTN1* | *TNFRSF6B* |
| *ANKRD46* | *CCND3* | *CXCL12* | *FLI1* | *ID4* | *LDHB* | *NFIB* | *PRDX5* | *SIGIRR* | *TNFRSF8* |
| *ANLN* | *CCNE1* | *CXCL13* | *FLNA* | *IDH1* | *LEF1* | *NFIL3* | *PRF1* | *SIGLEC1* | *TNFRSF9* |
| *ANO3* | *CCNE2* | *CXCL14* | *FLNB* | *IDH2* | *LEFTY1* | *NFKB1* | *PRG2* | *SIGLEC5* | *TNFSF10* |
| *ANP32B* | *CCNO* | *CXCL16* | *FLNC* | *IDO1* | *LEFTY2* | *NFKB2* | *PRICKLE1* | *SIGLEC8* | *TNFSF11* |
| *ANXA1* | *CCR1* | *CXCL2* | *FLT1* | *IDO2* | *LEP* | *NFKBIA* | *PRKAA2* | *SIL1* | *TNFSF12* |
| *AP1M1* | *CCR2* | *CXCL3* | *FLT3* | *IER3* | *LEPR* | *NFKBIE* | *PRKACA* | *SIN3A* | *TNFSF13* |
| *AP3B1* | *CCR3* | *CXCL5* | *FLT3LG* | *IFI16* | *LEXM* | *NFKBIZ* | *PRKACB* | *SIRPA* | *TNFSF13B* |
| *APAF1* | *CCR4* | *CXCL6* | *FLT4* | *IFI27* | *LFNG* | *NGF* | *PRKACG* | *SIRPB2* | *TNFSF14* |
| *APBB1* | *CCR5* | *CXCL8* | *FN1* | *IFI35* | *LGALS3* | *NGFR* | *PRKAR1A* | *SIRT4* | *TNFSF15* |
| *APC* | *CCR6* | *CXCL9* | *FOLH1* | *IFI44L* | *LGALS4* | *NID2* | *PRKAR1B* | *SIT1* | *TNFSF18* |
| *APC2* | *CCR7* | *CXCR1* | *FOS* | *IFI6* | *LGALS9* | *NKD1* | *PRKAR2A* | *SIX1* | *TNFSF4* |
| *APH1B* | *CCR9* | *CXCR2* | *FOSL1* | *IFIH1* | *LGR5* | *NKG7* | *PRKAR2B* | *SKAP2* | *TNFSF8* |
| *API5* | *CCRL2* | *CXCR3* | *FOXA1* | *IFIT1* | *LHX3* | *NKX2-1* | *PRKCA* | *SKP1* | *TNFSF9* |
| *APIP* | *CD101* | *CXCR4* | *FOXA2* | *IFIT2* | *LIF* | *NKX3-1* | *PRKCB* | *SKP2* | *TNKS* |
| *APLNR* | *CD14* | *CXCR5* | *FOXC1* | *IFIT3* | *LIFR* | *NLRC5* | *PRKCD* | *SLAMF1* | *TNN* |
| *APOA1* | *CD160* | *CXCR6* | *FOXE1* | *IFITM1* | *LIG1* | *NLRP3* | *PRKCE* | *SLAMF6* | *TNR* |
| *APOA2* | *CD163* | *CXXC4* | *FOXG1* | *IFITM2* | *LIG3* | *NOD1* | *PRKCG* | *SLAMF7* | *TOLLIP* |
| *APOA4* | *CD164* | *CXXC5* | *FOXJ1* | *IFNA1* | *LIG4* | *NOD2* | *PRKCQ* | *SLAMF8* | *TOP2A* |
| *APOB* | *CD180* | *CYB561* | *FOXL2* | *IFNA17* | *LILRA1* | *NODAL* | *PRKDC* | *SLC11A1* | *TOX* |
| *APOBEC3B* | *CD19* | *CYBB* | *FOXM1* | *IFNA2* | *LILRA4* | *NOG* | *PRKX* | *SLC12A7* | *TP53* |
| *APOC2* | *CD1A* | *CYCS* | *FOXO1* | *IFNA7* | *LILRA5* | *NOL4* | *PRL* | *SLC16A1* | *TP63* |
| *APOC3* | *CD1B* | *CYFIP2* | *FOXO3* | *IFNA8* | *LILRB1* | *NOL7* | *PRLR* | *SLC16A2* | *TP73* |
| *APOD* | *CD1C* | *CYLD* | *FOXO4* | *IFNAR1* | *LILRB2* | *NOP16* | *PRM1* | *SLC1A5* | *TPD52L1* |
| *APOE* | *CD1D* | *CYP17A1* | *FOXP3* | *IFNAR2* | *LILRB3* | *NOS1* | *PRMT8* | *SLC23A2* | *TPI1* |
| *APOL6* | *CD1E* | *CYP19A1* | *FPR1* | *IFNB1* | *LILRB4* | *NOS1AP* | *PROM1* | *SLC25A1* | *TPM1* |
| *APOLD1* | *CD2* | *CYP1B1* | *FPR2* | *IFNG* | *LIMA1* | *NOS2* | *PROS1* | *SLC26A4* | *TPM2* |
| *APOM* | *CD200* | *CYP2D6* | *FPR3* | *IFNGR1* | *LLGL1* | *NOS3* | *PRPF38A* | *SLC2A1* | *TPM3* |
| *APP* | *CD207* | *CYP4A11* | *FRAT1* | *IFNGR2* | *LMNA* | *NOTCH1* | *PRR5* | *SLC34A2* | *TPM4* |
| *APPL1* | *CD209* | *CYP4A22* | *FRAT2* | *IFNL1* | *LOH12CR1* | *NOTCH2* | *PRRX1* | *SLC35F2* | *TPO* |
| *AQP9* | *CD22* | *CYP8B1* | *FST* | *IFNL2* | *LOXL2* | *NOTCH3* | *PRSS1* | *SLC35F3* | *TPR* |
| *AR* | *CD226* | *CYSTM1* | *FSTL3* | *IGF1* | *LRG1* | *NOTCH4* | *PRUNE1* | *SLC39A6* | *TPSAB1* |
| *ARAF* | *CD24* | *DAB2* | *FUBP1* | *IGF1R* | *LRP1* | *NOX1* | *PSAT1* | *SLC3A1* | *TPSB2* |
| *AREG* | *CD244* | *DACH2* | *FUT4* | *IGF2* | *LRP2* | *NPM1* | *PSEN1* | *SLC3A2* | *TPTE* |
| *ARF1* | *CD247* | *DAPK1* | *FUT5* | *IGF2R* | *LRP5* | *NPM2* | *PSEN2* | *SLC43A1* | *TPX2* |
| *ARG1* | *CD27* | *DAPK2* | *FUT7* | *IGFBP2* | *LRP6* | *NPTX2* | *PSMB10* | *SLC43A2* | *TRAF1* |
| *ARG2* | *CD274* | *DAPK3* | *FUT8* | *IGFBP3* | *LRRC32* | *NPY1R* | *PSMB2* | *SLC45A3* | *TRAF2* |
| *ARHGEF2* | *CD276* | *DAXX* | *FYB1* | *IGFBP7* | *LRRC71* | *NQO1* | *PSMB3* | *SLC4A1AP* | *TRAF3* |
| *ARHGEF6* | *CD28* | *DCC* | *FYN* | *IGLL1* | *LRRN3* | *NR3C1* | *PSMB5* | *SLC4A4* | *TRAF4* |
| *ARID1A* | *CD300A* | *DCSTAMP* | *FZD1* | *IGSF6* | *LSM12* | *NR4A1* | *PSMB7* | *SLC4A7* | *TRAF5* |
| *ARID1B* | *CD33* | *DCTN1* | *FZD10* | *IHH* | *LSM14A* | *NR4A3* | *PSMB8* | *SLC5A5* | *TRAF6* |
| *ARID2* | *CD34* | *DDB1* | *FZD2* | *IKBKB* | *LST1* | *NRAP* | *PSMB9* | *SLC5A8* | *TRAF7* |
| *ARID5A* | *CD36* | *DDB2* | *FZD3* | *IKBKE* | *LTA* | *NRAS* | *PSMC4* | *SLC6A13* | *TRAK1* |
| *ARMC10* | *CD37* | *DDIT3* | *FZD4* | *IKBKG* | *LTB* | *NRBF2* | *PSMD7* | *SLC7A5* | *TRAT1* |
| *ARMH3* | *CD38* | *DDIT4* | *FZD5* | *IKZF1* | *LTBP1* | *NRDE2* | *PSPH* | *SLMAP* | *TREM1* |
| *ARNT* | *CD3D* | *DDX21* | *FZD6* | *IKZF2* | *LTBR* | *NRG1* | *PTCD2* | *SMAD2* | *TREM2* |
| *ARNT2* | *CD3E* | *DDX43* | *FZD7* | *IKZF3* | *LTF* | *NRG2* | *PTCH1* | *SMAD3* | *TRIM15* |
| *ARNTL* | *CD3G* | *DDX50* | *FZD8* | *IKZF4* | *LTK* | *NRG3* | *PTCH2* | *SMAD4* | *TRIM21* |
| *ASAP2* | *CD4* | *DDX58* | *FZD9* | *IL10* | *LUC7L2* | *NRP1* | *PTCRA* | *SMAD5* | *TRIM24* |
| *ASCL1* | *CD40* | *DEFB1* | *G6PD* | *IL10RA* | *LUM* | *NSD1* | *PTEN* | *SMAD9* | *TRIM27* |
| *ASL* | *CD40LG* | *DEFB134* | *GAB1* | *IL11* | *LY6E* | *NSD2* | *PTGDR2* | *SMAP1* | *TRIM29* |
| *ASNS* | *CD44* | *DEPTOR* | *GAB2* | *IL11RA* | *LY6K* | *NSD3* | *PTGDS* | *SMARCA2* | *TRIM33* |
| *ASPA* | *CD46* | *DGAT2* | *GABPA* | *IL12A* | *LY86* | *NT5E* | *PTGER4* | *SMARCA4* | *TRIM39* |
| *ASPG* | *CD47* | *DGCR2* | *GABRB2* | *IL12B* | *LY9* | *NTF3* | *PTGFRN* | *SMARCB1* | *TRIM63* |
| *ASPN* | *CD48* | *DGLUCY* | *GADD45A* | *IL12RB1* | *LY96* | *NTHL1* | *PTGS2* | *SMARCC1* | *TSC1* |
| *ASPSCR1* | *CD5* | *DHX15* | *GADD45B* | *IL12RB2* | *LYN* | *NTN3* | *PTK2* | *SMARCC2* | *TSC2* |
| *ASXL1* | *CD52* | *DHX16* | *GADD45G* | *IL13* | *LYZ* | *NTRK1* | *PTK7* | *SMARCD1* | *TSHR* |
| *ATF1* | *CD53* | *DIAPH1* | *GADD45GIP1* | *IL13RA1* | *LZTFL1* | *NTRK2* | *PTN* | *SMARCD2* | *TSLP* |
| *ATF2* | *CD55* | *DIO1* | *GADL1* | *IL13RA2* | *M6PR* | *NTRK3* | *PTPN11* | *SMARCD3* | *TSPAN7* |
| *ATF3* | *CD58* | *DIO2* | *GAGE1* | *IL15* | *MAD1L1* | *NUB1* | *PTPN5* | *SMARCE1* | *TSPAN8* |
| *ATF4* | *CD59* | *DIPK2B* | *GAGE10* | *IL15RA* | *MAD2L1* | *NUBP1* | *PTPN6* | *SMC1A* | *TTC30A* |
| *ATF7IP* | *CD6* | *DKK1* | *GAGE12F* | *IL16* | *MAD2L2* | *NUF2* | *PTPN7* | *SMC1B* | *TTC31* |
| *ATG10* | *CD63* | *DKK2* | *GAGE12I* | *IL17A* | *MADCAM1* | *NUMB* | *PTPRC* | *SMC3* | *TTK* |
| *ATG12* | *CD68* | *DKK4* | *GAGE12J* | *IL17B* | *MAF* | *NUMBL* | *PTPRCAP* | *SMO* | *TTPA* |
| *ATG16L1* | *CD69* | *DLK1* | *GAGE13* | *IL17F* | *MAFF* | *NUP107* | *PTPRD* | *SMPD3* | *TTR* |
| *ATG5* | *CD7* | *DLL1* | *GAGE2A* | *IL17RA* | *MAGEA1* | *NUP214* | *PTPRE* | *SNAI1* | *TUBB* |
| *ATG7* | *CD70* | *DLL3* | *GAGE2C* | *IL17RB* | *MAGEA10* | *NUPR1* | *PTPRN2* | *SNAI2* | *TUSC3* |
| *ATIC* | *CD74* | *DLL4* | *GAGE2E* | *IL18* | *MAGEA12* | *OAS1* | *PTPRR* | *SNCA* | *TWF1* |
| *ATM* | *CD79A* | *DLX2* | *GAPDH* | *IL18R1* | *MAGEA3* | *OAS2* | *PTPRZ1* | *SND1* | *TWIST1* |
| *ATOX1* | *CD79B* | *DMBT1* | *GAS1* | *IL18RAP* | *MAGEA4* | *OAS3* | *PTTG1* | *SOCS1* | *TWIST2* |
| *ATP11C* | *CD80* | *DNAJC14* | *GATA1* | *IL19* | *MAGEA6* | *OASL* | *PTTG2* | *SOCS2* | *TXK* |
| *ATP1B1* | *CD81* | *DNMT1* | *GATA2* | *IL1A* | *MAGEB2* | *OAT* | *PUM1* | *SOCS3* | *TXLNA* |
| *ATP2A2* | *CD83* | *DNMT3A* | *GATA3* | *IL1B* | *MAGEC1* | *OAZ1* | *PURA* | *SOD1* | *TXLNGY* |
| *ATP5F1D* | *CD84* | *DOCK9* | *GBP1* | *IL1R1* | *MAGEC2* | *OCIAD1* | *PVR* | *SOD2* | *TXN2* |
| *ATP5F1E* | *CD86* | *DPF1* | *GBP2* | *IL1R2* | *MAGI3* | *OFD1* | *PVRIG* | *SORBS1* | *TXNIP* |
| *ATP5ME* | *CD8A* | *DPF3* | *GBP4* | *IL1RAP* | *MALT1* | *OLFML2B* | *PWWP2A* | *SORBS2* | *TXNRD1* |
| *ATP6V1D* | *CD8B* | *DPP4* | *GCG* | *IL1RAPL2* | *MAML2* | *OLR1* | *PYCARD* | *SOS1* | *TXNRD2* |
| *ATR* | *CD9* | *DSC3* | *GCGR* | *IL1RL1* | *MAP2K1* | *OPN3* | *PYCR1* | *SOS2* | *TXNRD3* |
| *ATRX* | *CD96* | *DSP* | *GDF15* | *IL1RL2* | *MAP2K2* | *ORC6* | *PYCR2* | *SOST* | *TYK2* |
| *AURKA* | *CD99* | *DST* | *GDF6* | *IL1RN* | *MAP2K3* | *OSM* | *PYCR3* | *SOX10* | *TYMP* |
| *AURKB* | *CDC14A* | *DTX1* | *GEMIN4* | *IL2* | *MAP2K4* | *OTC* | *PYGL* | *SOX11* | *TYMS* |
| *AXIN1* | *CDC14B* | *DTX3* | *GFAP* | *IL20RA* | *MAP2K6* | *OTOA* | *QKI* | *SOX17* | *TYROBP* |
| *AXIN2* | *CDC20* | *DTX3L* | *GHITM* | *IL20RB* | *MAP3K1* | *OXR1* | *RAB3IL1* | *SOX2* | *TYRP1* |
| *AXL* | *CDC25A* | *DTX4* | *GHR* | *IL21* | *MAP3K12* | *P2RY13* | *RAB7A* | *SOX4* | *U2AF1* |
| *AZGP1* | *CDC25B* | *DUOX1* | *GIMAP4* | *IL21R* | *MAP3K13* | *P4HA1* | *RABGAP1L* | *SOX9* | *UBA7* |
| *B2M* | *CDC25C* | *DUOX2* | *GIMAP6* | *IL22* | *MAP3K14* | *P4HA2* | *RAC1* | *SP1* | *UBB* |
| *B3GAT1* | *CDC27* | *DUSP1* | *GIT2* | *IL22RA1* | *MAP3K20* | *PAK1* | *RAC2* | *SPA17* | *UBC* |
| *B4GALT6* | *CDC42* | *DUSP10* | *GJA1* | *IL22RA2* | *MAP3K5* | *PAK2* | *RAC3* | *SPACA3* | *UBE2C* |
| *BACH2* | *CDC42EP1* | *DUSP2* | *GJB6* | *IL23A* | *MAP3K7* | *PAK3* | *RAD18* | *SPAG17* | *UBE2T* |
| *BAD* | *CDC6* | *DUSP4* | *GKAP1* | *IL23R* | *MAP3K8* | *PAK4* | *RAD21* | *SPANXB1* | *ULBP2* |
| *BAG1* | *CDC7* | *DUSP5* | *GLI1* | *IL24* | *MAP4K2* | *PAK5* | *RAD23B* | *SPECC1L* | *UNC5D* |
| *BAG4* | *CDCA3* | *DUSP6* | *GLI2* | *IL25* | *MAPK1* | *PAK6* | *RAD50* | *SPI1* | *UNG* |
| *BAIAP2L1* | *CDH1* | *DUSP8* | *GLI3* | *IL26* | *MAPK10* | *PALMD* | *RAD51* | *SPIB* | *UPK1B* |
| *BAIAP3* | *CDH11* | *DVL1* | *GLIS3* | *IL27* | *MAPK11* | *PAN3* | *RAD51C* | *SPINK1* | *UPK3A* |
| *BAK1* | *CDH16* | *DVL2* | *GLOD4* | *IL2RA* | *MAPK12* | *PANX3* | *RAD52* | *SPINK5* | *UQCR10* |
| *BAMBI* | *CDH17* | *DVL3* | *GLS* | *IL2RB* | *MAPK14* | *PAPD7* | *RAD54L* | *SPINT1* | *UQCR11* |
| *BAP1* | *CDH2* | *DYNC1I2* | *GLUD1* | *IL2RG* | *MAPK3* | *PAPSS1* | *RAF1* | *SPN* | *UQCRQ* |
| *BATF* | *CDH3* | *DZANK1* | *GLUL* | *IL3* | *MAPK8* | *PARG* | *RAG1* | *SPO11* | *USP10* |
| *BATF3* | *CDH5* | *E2F1* | *GMIP* | *IL32* | *MAPK8IP1* | *PARP12* | *RALA* | *SPOCK2* | *USP39* |
| *BAX* | *CDK1* | *E2F2* | *GNA11* | *IL33* | *MAPK8IP2* | *PARP2* | *RALB* | *SPOP* | *USP8* |
| *BBC3* | *CDK12* | *E2F3* | *GNA14* | *IL34* | *MAPK9* | *PARP4* | *RALBP1* | *SPP1* | *USP9Y* |
| *BBS1* | *CDK2* | *E2F4* | *GNAQ* | *IL3RA* | *MAPKAPK2* | *PARP9* | *RALGDS* | *SPRED1* | *UST* |
| *BCAN* | *CDK4* | *E2F5* | *GNAS* | *IL4* | *MAPT* | *PASD1* | *RANBP2* | *SPRED2* | *UTY* |
| *BCAT1* | *CDK6* | *EBI3* | *GNG12* | *IL4R* | *MARCKS* | *PAWR* | *RAP1A* | *SPRY1* | *VCAM1* |
| *BCL10* | *CDKN1A* | *ECSIT* | *GNG4* | *IL5* | *MARCO* | *PAX3* | *RAP1B* | *SPRY2* | *VCAN* |
| *BCL11B* | *CDKN1B* | *EDC3* | *GNG7* | *IL5RA* | *MASP1* | *PAX5* | *RAPGEF1* | *SPRY4* | *VCL* |
| *BCL2* | *CDKN1C* | *EDN1* | *GNGT1* | *IL6* | *MASP2* | *PAX8* | *RARA* | *SQSTM1* | *VEGFA* |
| *BCL2A1* | *CDKN2A* | *EEF1G* | *GNL3* | *IL6R* | *MAVS* | *PBK* | *RARB* | *SRC* | *VEGFB* |
| *BCL2L1* | *CDKN2B* | *EFNA1* | *GNLY* | *IL6ST* | *MAX* | *PBRM1* | *RASA4* | *SRD5A2* | *VEGFC* |
| *BCL2L11* | *CDKN2C* | *EFNA2* | *GOLGA5* | *IL7* | *MBL2* | *PBX1* | *RASAL1* | *SREBF1* | *VEGFD* |
| *BCL2L14* | *CDKN2D* | *EFNA3* | *GOPC* | *IL7R* | *MBNL1* | *PBX3* | *RASGEF1B* | *SRGN* | *VHL* |
| *BCL3* | *CDKN3* | *EFNA4* | *GOT1* | *IL9* | *MBNL3* | *PC* | *RASGRF1* | *SRP54* | *VIM* |
| *BCL6* | *CDX2* | *EFNA5* | *GOT2* | *ILF3* | *MCAM* | *PCBP1* | *RASGRF2* | *SRR* | *VOPP1* |
| *BCL6B* | *CEACAM1* | *EGF* | *GPATCH3* | *ILK* | *MCAT* | *PCDH7* | *RASGRP1* | *SRSF2* | *VPS33B* |
| *BCOR* | *CEACAM3* | *EGFR* | *GPC4* | *ING4* | *MCL1* | *PCK1* | *RASGRP2* | *SS18* | *VSIR* |
| *BCR* | *CEACAM5* | *EGLN1* | *GPI* | *INHBA* | *MCM2* | *PCK2* | *RASSF1* | *SSBP1* | *VSTM2A* |
| *BDNF* | *CEACAM6* | *EGLN2* | *GPM6B* | *INHBB* | *MCM4* | *PCLAF* | *RASSF5* | *SSBP2* | *VTCN1* |
| *BGN* | *CEACAM8* | *EGLN3* | *GPR160* | *INPP5D* | *MCM5* | *PCM1* | *RB1* | *SST* | *WAC* |
| *BICC1* | *CEBPA* | *EGR1* | *GPR18* | *INS* | *MCM7* | *PCNA* | *RBL2* | *SSX1* | *WDCP* |
| *BID* | *CEBPB* | *EGR2* | *GPR3* | *INSL4* | *MDC1* | *PCP4* | *RBM45* | *SSX2* | *WDR3* |
| *BIRC2* | *CEBPE* | *EGR3* | *GPS1* | *INSRR* | *MDFIC* | *PDCD1* | *RBMS3* | *SSX4* | *WDR76* |
| *BIRC3* | *CELSR2* | *EHHADH* | *GPSM3* | *IRAK1* | *MDM2* | *PDCD1LG2* | *RBP4* | *ST6GAL1* | *WEE1* |
| *BIRC5* | *CENPF* | *EIF1* | *GPT* | *IRAK2* | *MDM4* | *PDE5A* | *RBPMS* | *ST7* | *WIF1* |
| *BIRC7* | *CEP43* | *EIF2AK2* | *GPX1* | *IRAK3* | *ME2* | *PDE7A* | *RBX1* | *STAG2* | *WIPF1* |
| *BLK* | *CEP55* | *EIF2AK3* | *GPX3* | *IRAK4* | *MECOM* | *PDE9A* | *RCC1* | *STARD3* | *WIPF2* |
| *BLM* | *CEP72* | *EIF2B4* | *GPX4* | *IRF1* | *MED12* | *PDGFA* | *REG4* | *STAT1* | *WNK2* |
| *BLNK* | *CEP85L* | *EIF3L* | *GRAP2* | *IRF2* | *MEF2C* | *PDGFB* | *REL* | *STAT2* | *WNT1* |
| *BLVRA* | *CEP89* | *EIF4A2* | *GRB10* | *IRF2BP2* | *MEF2D* | *PDGFC* | *RELA* | *STAT3* | *WNT10A* |
| *BMI1* | *CES3* | *EIF4EBP1* | *GRB2* | *IRF3* | *MEFV* | *PDGFD* | *RELB* | *STAT4* | *WNT10B* |
| *BMP2* | *CFB* | *EIF5AL1* | *GRB7* | *IRF4* | *MEIS1* | *PDGFRA* | *RELN* | *STAT5A* | *WNT11* |
| *BMP4* | *CFD* | *ELANE* | *GREM1* | *IRF5* | *MELK* | *PDGFRB* | *REN* | *STAT5B* | *WNT16* |
| *BMP5* | *CFI* | *ELAVL3* | *GRIA3* | *IRF7* | *MEN1* | *PDK1* | *REPS1* | *STAT6* | *WNT2* |
| *BMP6* | *CFL1* | *ELAVL4* | *GRIN1* | *IRF8* | *MERTK* | *PDLIM4* | *RET* | *STC1* | *WNT2B* |
| *BMP7* | *CFP* | *ELK1* | *GRIN2A* | *IRF9* | *MET* | *PDPK1* | *REV1* | *STING1* | *WNT3* |
| *BMP8A* | *CGAS* | *ELMO1* | *GRIN2B* | *IRGM* | *MFGE8* | *PDPN* | *REV3L* | *STK11* | *WNT3A* |
| *BMPR1B* | *CHAD* | *ELOB* | *GRIPAP1* | *IRS1* | *MFNG* | *PDZK1IP1* | *RFC3* | *STK11IP* | *WNT4* |
| *BNIP3* | *CHEK1* | *ELOC* | *GSK3B* | *ISG15* | *MGEA5* | *PEAR1* | *RFC4* | *STK17B* | *WNT5A* |
| *BNIP3L* | *CHEK2* | *ELOVL6* | *GSN* | *ISG20* | *MGMT* | *PEBP1* | *RGMB* | *STK26* | *WNT5B* |
| *BRAF* | *CHGA* | *EML4* | *GSTA1* | *ISL1* | *MGP* | *PECAM1* | *RGS17* | *STK4* | *WNT6* |
| *BRCA1* | *CHI3L1* | *EMX2* | *GSTA2* | *ITCH* | *MGST1* | *PEG3* | *RHOA* | *STMN1* | *WNT7A* |
| *BRCA2* | *CHIT1* | *ENDOG* | *GSTA3* | *ITGA1* | *MGST2* | *PER2* | *RHOB* | *STMN2* | *WNT7B* |
| *BRD2* | *CHRM3* | *ENG* | *GSTA4* | *ITGA2* | *MGST3* | *PF4* | *RICTOR* | *STON1-GTF2A1L* | *WNT8A* |
| *BRD3* | *CHSY1* | *ENO1* | *GSTA5* | *ITGA2B* | *MIA* | *PFKFB3* | *RIMKLA* | *STRN* | *WNT8B* |
| *BRD4* | *CHTOP* | *ENTPD1* | *GSTM1* | *ITGA3* | *MIB1* | *PFKM* | *RIMKLB* | *STRN3* | *WNT9A* |
| *BRD7* | *CHUK* | *EOMES* | *GSTM2* | *ITGA4* | *MICA* | *PGAP3* | *RIN1* | *SUFU* | *WNT9B* |
| *BRIP1* | *CIC* | *EP300* | *GSTM3* | *ITGA5* | *MICB* | *PGF* | *RIPK1* | *SULF1* | *WRN* |
| *BRIX1* | *CIDEA* | *EPAS1* | *GSTM4* | *ITGA6* | *MIF* | *PGK1* | *RIPK2* | *SULT2A1* | *WT1* |
| *BST1* | *CIITA* | *EPCAM* | *GSTM5* | *ITGA7* | *MITF* | *PGM2* | *RIPK3* | *SUMO1* | *WWC1* |
| *BST2* | *CIT* | *EPHA2* | *GSTO1* | *ITGA8* | *MKI67* | *PGPEP1* | *RNF130* | *SUV39H2* | *XAGE1B* |
| *BTBD1* | *CKLF* | *EPM2AIP1* | *GSTO2* | *ITGA9* | *MKRN1* | *PGR* | *RNF213* | *SYCP1* | *XCL1* |
| *BTF3L4* | *CKS1B* | *EPO* | *GSTP1* | *ITGAE* | *MLANA* | *PHC3* | *RNF43* | *SYK* | *XCL2* |
| *BTK* | *CKS2* | *EPOR* | *GSTT1* | *ITGAL* | *MLEC* | *PHF10* | *RNF8* | *SYT12* | *XCR1* |
| *BTLA* | *CLCF1* | *EPS15* | *GSTT2* | *ITGAM* | *MLF1* | *PHF12* | *RNLS* | *SYT17* | *XIAP* |
| *BUB1* | *CLCN6* | *EPS8L3* | *GSTT2B* | *ITGAV* | *MLH1* | *PHF6* | *ROBO4* | *TAB1* | *XIST* |
| *BUB1B-PAK6* | *CLDN18* | *ERBB2/HER2* | *GTF2H3* | *ITGAX* | *MLLT10* | *PHGDH* | *ROCK1* | *TACC1* | *XPA* |
| *BYSL* | *CLEC10A* | *ERBB4* | *GTF2I* | *ITGB1* | *MLLT3* | *PHLDA2* | *ROPN1* | *TACC2* | *XRCC2* |
| *C1QA* | *CLEC14A* | *ERC1* | *GTF2IRD1* | *ITGB2* | *MLPH* | *PHLDB3* | *ROR2* | *TACC3* | *XRCC4* |
| *C1QB* | *CLEC4A* | *ERCC1* | *GTF3C1* | *ITGB3* | *MME* | *PI15* | *RORA* | *TACSTD2* | *XRCC5* |
| *C1QBP* | *CLEC4C* | *ERCC2* | *GTPBP4* | *ITGB4* | *MMP1* | *PIAS1* | *RORC* | *TAF3* | *XRCC6* |
| *C1R* | *CLEC4E* | *ERCC3* | *GUSB* | *ITGB6* | *MMP11* | *PIAS2* | *ROS1* | *TAGAP* | *XXYLT1* |
| *C1S* | *CLEC5A* | *ERCC4* | *GYG1* | *ITGB7* | *MMP12* | *PIAS3* | *RPA3* | *TAL1* | *YRDC* |
| *C2* | *CLEC6A* | *ERCC5* | *GZMA* | *ITGB8* | *MMP2* | *PIAS4* | *RPL23* | *TANK* | *YTHDF2* |
| *C2CD5* | *CLEC7A* | *ERCC6* | *GZMB* | *ITK* | *MMP3* | *PIGR* | *RPL3* | *TAP1* | *YWHAE* |
| *C3* | *CLECL1* | *EREG* | *GZMH* | *ITPK1* | *MMP7* | *PIK3CA* | *RPL4* | *TAP2* | *ZAN* |
| *C3AR1* | *CLIP1* | *ERG* | *GZMK* | *JADE1* | *MMP9* | *PIK3CB* | *RPL7A* | *TAPBP* | *ZAP70* |
| *C4B* | *CLTC* | *ERLIN2* | *GZMM* | *JAG1* | *MMRN2* | *PIK3CD* | *RPLP0* | *TAPBPL* | *ZBTB16* |
| *C4BPA* | *CLU* | *ERN2* | *H2AX* | *JAG2* | *MNAT1* | *PIK3CG* | *RPS11* | *TARP* | *ZBTB17* |
| *C5* | *CMA1* | *ERO1A* | *H3-3A* | *JAK1* | *MNX1* | *PIK3R1* | *RPS14* | *TATDN1* | *ZBTB20* |
| *C5AR1* | *CMKLR1* | *ERP44* | *H3-5* | *JAK2* | *MORC3* | *PIK3R2* | *RPS27A* | *TAX1BP1* | *ZBTB32* |
| *C6* | *CMTM4* | *ESR1* | *H3C10* | *JAK3* | *MPL* | *PIK3R3* | *RPS4Y1* | *TBC1D1* | *ZBTB46* |
| *C7* | *CMTM6* | *ESR2* | *H3C2* | *JAKMIP1* | *MPO* | *PIK3R4* | *RPS6* | *TBC1D10B* | *ZC3H12A* |
| *C8A* | *CNIH4* | *ETHE1* | *H3C8* | *JAM3* | *MPPED1* | *PIK3R5* | *RPS6KA5* | *TBC1D2* | *ZC3H14* |
| *C8B* | *CNOT10* | *ETS1* | *HACD2* | *JAML* | *MPRIP* | *PIM1* | *RPS6KA6* | *TBK1* | *ZC3HAV1* |
| *C8G* | *CNOT2* | *ETS2* | *HAMP* | *JCHAIN* | *MR1* | *PIM2* | *RPS6KB1* | *TBL1XR1* | *ZCCHC8* |
| *C8orf34* | *CNOT4* | *ETV1* | *HAVCR2* | *JUN* | *MRC1* | *PIN1* | *RPS6KB2* | *TBP* | *ZEB1* |
| *C9* | *CNTFR* | *ETV4* | *HBB* | *JUNB* | *MRE11* | *PITX2* | *RPS9* | *TBX21* | *ZEB2* |
| *CA12* | *CNTRL* | *ETV5* | *HBEGF* | *JUP* | *MRM2* | *PKM* | *RPTOR* | *TBXAS1* | *ZIC2* |
| *CA2* | *COG7* | *ETV6* | *HCK* | *KAT2B* | *MRPL19* | *PKMYT1* | *RRAD* | *TCF3* | *ZKSCAN5* |
| *CA4* | *COL11A1* | *ETV7* | *HDAC1* | *KATNAL2* | *MRPS5* | *PKP3* | *RRAS2* | *TCF7* | *ZMYM2* |
| *CACNA1C* | *COL11A2* | *EVA1A* | *HDAC10* | *KBTBD8* | *MS4A1* | *PLA1A* | *RRM2* | *TCF7L1* | *ZMYM4* |
| *CACNA1D* | *COL14A1* | *EWSR1* | *HDAC11* | *KCNAB1* | *MS4A2* | *PLA2G10* | *RRS1* | *TCF7L2* | *ZNF143* |
| *CACNA1E* | *COL16A1* | *EXO1* | *HDAC2* | *KCNIP3* | *MS4A4A* | *PLA2G1B* | *RSAD2* | *TCIM* | *ZNF205* |
| *CACNA1G* | *COL17A1* | *EYA1* | *HDAC3* | *KCNJ11* | *MS4A6A* | *PLA2G2A* | *RSPH14* | *TCL1A* | *ZNF34* |
| *CACNA1H* | *COL1A1* | *EZH2* | *HDAC4* | *KCNN4* | *MSH2* | *PLA2G3* | *RTN4RL1* | *TCL1B* | *ZNF346* |
| *CACNA2D1* | *COL1A2* | *EZR* | *HDAC5* | *KCTD8* | *MSH3* | *PLA2G4A* | *RUNX1* | *TDO2* | *ZNF365* |
| *CACNA2D2* | *COL24A1* | *F11* | *HDAC6* | *KDELR2* | *MSH6* | *PLA2G4C* | *RUNX1T1* | *TEAD2* | *ZNF384* |
| *CACNA2D3* | *COL27A1* | *F11R* | *HDC* | *KDM5C* | *MSMB* | *PLA2G4E* | *RUNX2* | *TECR* | *ZNF485* |
| *CACNA2D4* | *COL2A1* | *F12* | *HELLS* | *KDM6A* | *MSN* | *PLA2G4F* | *RUNX3* | *TERC* | *ZNF703* |
| *CACNB2* | *COL3A1* | *F13A1* | *HERC6* | *KDM7A* | *MSR1* | *PLA2G5* | *RXRA* | *TERF2* | *ZSCAN30* |

**Table S6.** Clinicopathological characteristics of TNBC patients with different copy number subgroups (*n* = 178). Data are presented as number (percentage) or median (range).

|  | **Group 1** | **Group 2** | **Group 3** | **Group 4** | ***p* value** |
| --- | --- | --- | --- | --- | --- |
|  | **(*n =* 15)** | **(*n =* 4)** | **(*n =* 30)** | **(*n =* 129)** |  |
| Age (range), yrs | 48 (33–64) | 53.5 (47–61) | 47 (27–78) | 52 (26–87) | 0.527 |
| Menopause |  |  |  |  | 0.600 |
| Pre- and peri- | 9 (60.0%) | 2 (50.0%) | 15 (50.0%) | 56 (43.4%) |  |
| Post- | 6 (40.0%) | 2 (50.0%) | 15 (50.0%) | 73 (56.6%) |  |
| AJCC stage |  |  |  |  | 0.471 |
| I | 8 (53.3%) | 3 (75.0%) | 10 (33.3%) | 56 (43.4%) |  |
| II | 4 (26.7%) | 0 (0) | 14 (46.7%) | 56 (43.4%) |  |
| III | 3 (20.0%) | 1 (25.0%) | 5 (16.7%) | 14 (10.9%) |  |
| IV | 0 (0) | 0 (0) | 1 (3.3%) | 2 (1.6%) |  |
| Unknown | 0 (0) | 0 (0) | 0 (0) | 1 (0.8%) |  |
| Histology* |  |  |  |  | 0.814 |
| IBC-NST | 14 (93.3%) | 5 (100.0%) | 31 (96.9%) | 122 (93.8%) |  |
| Other | 1 (6.7%) | 0 (0) | 1 (3.1%) | 8 (6.2%) |  |
| Histological grade* |  |  |  |  | 0.239 |
| Grade 1 | 1 (6.7%) | 0 (0) | 0 (0) | 3 (2.3%) |  |
| Grade 2 | 2 (13.3%) | 1 (20.0%) | 4 (12.5%) | 32 (24.6%) |  |
| Grade 3 | 12 (80.0%) | 3 (60.0%) | 26 (81.3%) | 92 (70.8%) |  |
| Unknown | 0 (0) | 1 (20.0%) | 2 (6.3%) | 3 (2.3%) |  |
| HER2 expression* |  |  |  |  | 0.921 |
| Low | 6 (40.0%) | 2 (40.0%) | 14 (43.8%) | 62 (47.7%) |  |
| Zero | 9 (60.0%) | 3 (60.0%) | 18 (56.3%) | 68 (52.3%) |  |
| Ki-67 index (range), %* | 80 (10–90) | 70 (50–90) | 70 (10–95) | 60 (5–90) | 0.043 |
| Death |  |  |  |  | 0.851 |
| Present | 1 (6.7%) | 0 (0) | 1 (3.3%) | 12 (9.3%) |  |
| Absent | 14 (93.3%) | 4 (100.0%) | 29 (96.7%) | 117 (90.7%) |  |
| Other malignancies |  |  |  |  | 0.423 |
| Present | 1 (6.7%) | 0 (0) | 2 (6.7%) | 22 (17.1%) |  |
| Absent | 14 (93.3%) | 4 (100.0%) | 28 (93.3%) | 107 (82.9%) |  |
| Family history of malignancies |  |  |  |  | 0.471 |
| Breast cancer | 2 (13.3%) | 1 (25.0%) | 2 (6.7%) | 13 (10.1%) |  |
| Other malignancies | 3 (20.0%) | 0 (0) | 7 (23.3%) | 16 (12.4%) |  |
| Absent | 10 (66.7%) | 3 (75.0%) | 21 (70.0%) | 100 (77.5%) |  |

*The majority of the clinical data were analysed at the patient level, whereas tumour-specific characteristics including histological type, tumour grade, HER2 status, and Ki-67 index were assessed on a per-tumour basis.

TNBC, triple-negative breast cancer; FISH, fluorescence *in situ* hybridization.
